# Identification of DUSP4/6 overexpression as a potential rheostat to NRAS-induced hepatocarcinogenesis

**DOI:** 10.1186/s12885-023-11577-9

**Published:** 2023-11-09

**Authors:** Sophie Klemm, Katja Evert, Kirsten Utpatel, Alexandra Muggli, Maria M. Simile, Xin Chen, Matthias Evert, Diego F. Calvisi, Alexander Scheiter

**Affiliations:** 1 Institute of Pathology, University of Regensburg, Regensburg, Germany; 2 Department of Medicine, Surgery, and Pharmacy, University of Sassari, Sassari, Italy; 3 Cancer Biology Program, University of Hawaii Cancer Center, Honolulu, HI USA

**Keywords:** Hepatocellular carcinoma, Cholangiocarcinoma, NRAS, DUSP4, CD133, MAPK pathway

## Abstract

**Background:**

Upregulation of the mitogen-activated protein kinase (MAPK) cascade is common in hepatocellular carcinoma (HCC). Neuroblastoma RAS viral oncogene homolog (*NRAS*) is mutated in a small percentage of HCC and is hitherto considered insufficient for hepatocarcinogenesis. We aimed to characterize the process of N-Ras-dependent carcinogenesis in the liver and to identify potential therapeutic vulnerabilities.

**Methods:**

*NRAS* V12 plasmid was delivered into the mouse liver via hydrodynamic tail vein injection (HTVI). The resulting tumours, preneoplastic lesions, and normal tissue were characterized by NanoString® gene expression analysis, Western Blot, and Immunohistochemistry (IHC). The results were further confirmed by in vitro analyses of HCC cell lines.

**Results:**

HTVI with *NRAS* V12 plasmid resulted in the gradual formation of preneoplastic and neoplastic lesions in the liver three months post-injection. These lesions mostly showed characteristics of HCC, with some exceptions of spindle cell/ cholangiocellular differentiation. Progressive upregulation of the RAS/RAF/MEK/ERK signalling was detectable in the lesions by Western Blot and IHC. NanoString® gene expression analysis of preneoplastic and tumorous tissue revealed a gradual overexpression of the cancer stem cell marker CD133 and Dual Specificity Phosphatases 4 and 6 (DUSP4/6). In vitro, transfection of HCC cell lines with *NRAS* V12 plasmid resulted in a coherent upregulation of DUSP4 and DUSP6. Paradoxically, this upregulation in PLC/PRF/5 cells was accompanied by a downregulation of phosphorylated extracellular-signal-regulated kinase (pERK), suggesting an overshooting compensation. Silencing of DUSP4 and DUSP6 increased proliferation in HCC cell lines.

**Conclusions:**

Contrary to prior assumptions, the G12V *NRAS* mutant form is sufficient to elicit hepatocarcinogenesis in the mouse. Furthermore, the upregulation of the MAPK cascade was paralleled by the overexpression of DUSP4, DUSP6, and CD133 in vivo and in vitro. Therefore, DUSP4 and DUSP6 might fine-tune the excessive MAPK activation, a mechanism that can potentially be harnessed therapeutically.

**Supplementary Information:**

The online version contains supplementary material available at 10.1186/s12885-023-11577-9.

## Background

Primary liver cancer is the third leading cause of cancer-related deaths worldwide. It comprises both hepatocellular carcinoma (HCC) (~ 80% of cases) and intrahepatic cholangiocarcinoma (iCCA; ~ 15%), as well as other rare cancer types [1]. Both entities are often detected late, have a dismal prognosis, and have limited systemic treatment options [2, 3]. HCC and iCCA are molecularly very distinct entities: while in iCCA, driver mutations in oncogenes, including Kirsten rat sarcoma virus (*KRAS*), are frequently reported [4], in HCC *RAS* and rapidly accelerated fibrosarcoma (*RAF)* mutations occur only in a subfraction of less than 5 percent of cases [5]. Nonetheless, the activation of the RAS/MEK/ERK signalling cascade plays a pivotal role in HCC, given that it is often found to be strongly activated [6, 7]. In addition, the increased expression of RAS, Mitogen-activated protein kinase kinase (MEK), and ERK confers a dismal prognosis in HCC patients [8]. Mechanistically, indirect modalities of activation have been defined, which comprise the upregulation of positive modulators of RAS (such as guanine nucleotide exchange factor [9]) and the downregulation of RAS repressors (GTPase-activating proteins, Sprouty, etc. [10]). Moreover, paracrine stimulation by the hepatocyte growth factor (HGF), the c-Met ligand, represents another mode of RAS signalling activation [11]. The contribution of the RAS/MEK/ERK pathway to hepatocarcinogenesis has been studied extensively using the hydrodynamic tail vein injection (HTVI) technique. Surprisingly, the administration of mutated Ras homologues alone was insufficient to elicit tumorigenesis in the liver of immune-competent mice. This event was attributed to the induction of senescence and CD4^+^ T-cell-mediated clearing of pre-malignant hepatocytes by *NRAS*^G12V^ [12]. However, Ho et al. [13] demonstrated that adding myristoylated AKT to *NRAS*^G12V^ resulted in strong cooperativity and the development of HCC with a latency of only 4 weeks. Other studies have since confirmed the requirement of such cooperativity with observed tumorigenesis in *HRAS* + *MYC* [14], *RAS* + *Bmi1* [15], *NRAS* + *∆N90-β-catenin* co-injection models [5]. In one report, a weak hepatocarcinogenesis with the appearance of HCC by 5 months was also reported following the administration of HRAS^G12V^ alone, which the author attributed to this particular RAS isoform [16]. Of note, the authors did not find signs of senescence in this model.

The current work was originally aimed at examining the effect of *Rassf1a* knockout on *NRAS*-induced carcinogenesis. *RASSF1A* is considered a negative feedback regulator of *RAS* [17]. *RASSF1A* has been implied as a critical tumour suppressor in human hepatocarcinogenesis, where hypermethylation and inactivation of this gene frequently occur [18]. In accordance with our previous study, which did not show cooperativity with activating *PIK3CA* mutations [19], we could not find an increase in tumorigenesis resulting from *NRAS*^G12V^ injections. Despite this, we were intrigued by the observation that the injection of *NRAS*^G12V^ alone was sufficient to induce liver tumours (both in *Rassf1a* wildtype and knockout mice) after a latency as short as 3 months. Consequently, the previously described mechanisms of senescence inductions are insufficient to inhibit tumorigenesis in our model, and the unique opportunity of studying the oncogenesis of predominantly RAS/MAPK-driven HCC unfolded.

Of particular interest was the concomitant upregulation of the dual specificity phosphatases 4 and 6 (DUSP4 and 6), whose inactivation has recently emerged as digenic synthetic lethal targets in *NRAS* and *BRAF* mutant melanoma cell lines acting through ERK hyperactivation [20]. DUSP proteins can dephosphorylate and inactivate ERK1/2 proteins and are involved in the negative regulation of RAS/MAPK signalling [21, 22]. Of note, it has previously been hypothesized that DUSPs can restrain excessive RAS signalling, support oncogenic transformation, and counteract the induction of senescence [21, 23, 24]. Moreover, the strong upregulation of the cancer stem cell marker CD133 was determined as a defining feature already at an early time point of *RAS*-dependent hepatocarcinogenesis. This protein has recently been implicated in remedying defects in proliferative signalling [25]. Cell culture experiments unveiled a clear transcriptional dependence of the latter genes on RAS hyperactivation, and upregulation of DUSP4, DUSP6, and CD133 was confirmed both in HCC and CCA cell lines. Thus, novel features of *RAS* dependent-oncogenesis were uncovered, potentially representing therapeutic targets in the important oncological paradigm of *RAS*-dependent cancers.

## Methods

### Constructs and reagents

The original pT/Caggs-V12Nras [26] had been cloned into the pT3 backbone to generate pT3-CAGGS-NRasV12 [27]. pT3-CAGGS-NRasV12 was applied with pPGK-SB13 for HTVI as described previously [27]. The pT3-CAGGS-NRasV12 vector was furthermore used for the transfection of HCC cell lines. pT3-EF1α constituted the empty vector (EV) control. The control group's data have been published previously [19]. Purification of the plasmids was performed with the Endotoxin-free Maxi Prep Kit (Sigma-Aldrich, St.Louis, MO).

### Mouse breeding and genotyping

A *Rassf1a* KO founder breeding pair was kindly provided by Dr. Louise van der Weyden (Wellcome Trust Sanger Institute, Research Support Facility, Hinxton, Cambridge, CB10 1SA, UK). Note that the genetic background of *Rassf1a* WT and KO mice was C57BL/6 J x 129 Sv. As previously described [19], the control group of *Rassf1a* WT mice was generated by crossing *Rassf1a* KO mice with C57BL/6 J mice purchased from Charles River Laboratories (Sandhofer Weg, 97,633 Sulzfeld, Germany). Breeding conditions and PCR verification were identical to our report of *Rassf1a* KO with *PIK3CA* injections [19].

### Hydrodynamic injections and tissue collection

We followed exactly the previously described protocol [19]. Mice with an age of 6–8 weeks were subjected to HTVI. The experimental groups comprised: untreated, 1 × phosphate-buffered saline (PBS), empty vector (EV), and pT/Caggs-V12Nras co-injected with pPGK-SB13 in a ratio of 25 to 1 in a total volume of 2 ml 0.9% sodium chloride. The total volume was injected into the lateral tail vein within 5 to 7 s, resulting in temporal right-sided heart failure and increased hydrodynamic pressure in the hepatic veins. The distribution of mice per group is summarized in Supplementary Table 1.

The quality of the injection was defined by the following criteria, which led to the exclusion of the respective mice from further analysis: less than 2 ml of total injection and subsequent unusual behaviour lacking the expected reduction in the movement for at least 60 min. Termination criteria were palpable masses (equivalent to a tumour diameter of about 4 cm), respiratory distress, and lethargy. Animals were euthanized by cervical dislocation, subjected to a standardized autopsy protocol, and livers were photo-documented. Animal breeding and animal experiments were in accordance with protocols by the Mecklenburg-Western Pomeranian federal institution “Landesamt für Landwirtschaft, Lebensmittelsicherheit und Fischerei (LALLF) Mecklenburg-Vorpommern” (protocol number/Aktenzeichen: 7221.3–1.1–052/12).

### Cell culture and in vitro experiments

The human HCC cell lines PLC/PRF/5, HLE, HLF, Snu182, Snu387, Snu449, Hep3b, SK-HEP1, MHCC97-L, HuH6, as well as the human hepatoblastoma cell line Hep-G2 and the human cholangiocarcinoma cell lines KKU-M055, KKU-100, KKU213, HuCC-A1, CC-LP-1, OZ, RBE, SG231, TKKK, and YSCCC were cultured in 5% CO_2_ at 37 °C in a humidified incubator. Cell lines were purchased from ATCC (Manassas, Virginia, USA). Cells were grown in Dulbecco’s modified Eagle medium (Gibco, Grand Island, NY) or RPMI 1640 Medium (Gibco) supplemented with 5% fetal bovine serum (Gibco), 100 mg/mL streptomycin, and 100 U/mL penicillin, 1% 1 M HEPES Buffer (Gibco), 1% 100 mM sodium pyruvate (Gibco) and 1% GlutaMAX™ (Gibco).

The AKT/NRAS cell line was derived from a murine HCC that developed after HTVI of myristoylated AKT^flox/flox^ and *NRAS*^G12V^ [27]. Transfection of Cre recombinase resulted in the AKT Cre/NRAS cell line, which was driven solely by the *NRAS*^G12V^ oncogene, while AKT was depleted. These cells were maintained in Dulbecco’s modified Eagle medium (Gibco, Grand Island, NY).

For DUSP4 silencing, we seeded cells at a density of 4 × 10^5^ cells in 2 ml of medium per well in 6 well plates. Cells were transfected either with DUSP4 and NEG siPOOL (Catalogue numbers: Dusp4-m-002 and N000-c1-059; siTOOLs Biotech GmbH, Planegg, Germany) using Lipofectamine® RNAiMAX (Thermo Fisher Inc). Lipofectamine and siRNA were diluted and combined in OptiMEM® Reduced Serum Medium (Thermo Fisher Inc.). After an incubation period of 48 h, the transfection was repeated. Subsequently, the cells were harvested after an additional 24 h of incubation with cell scrapers. Cells were pelleted by centrifugation (300 g, 5 min). These pellets were used for further downstream analyses.

For transfections, cells were inspected for viability and confluency of about 70–80% and seeded at a density of 4 × 10^5^ cells in 2 mL of medium per well in a 6-well plate. Cells were transfected with pT3-CAGGS-NRasV12 or empty pT3 vector the following day using Lipofectamine® 2000. Lipofectamine and vectors were diluted in OptiMEM® Reduced Serum Medium and combined. Medium in the wells was discarded, and cells were washed with 1xPBS before the addition of the transfection medium. Cells were harvested and pelleted by centrifugation after 48 h incubation using cell scrapers.

For MEK-inhibition experiments, trametinib (Selleck Chemicals GmbH, Berlin, Germany) and mirdametinib (Selleck Chemicals GmbH) were applied. Cells were seeded in 6 well plates at a density of 3–5 × 10^5^ cells in 2 mL medium per well. The control group was treated with DMSO (concentration matching the highest inhibitor concentration as all inhibitors were dissolved in DMSO). After incubation for 24 h at the indicated concentrations, cells were harvested, and cell pellets were obtained.

TPA (12-O-Tetradecanoylphorbol-13-acetate; P1585, Sigma-Aldrich), a phorbol ester, which induces pERK1/2 via the protein kinase C (PKC) [28], was administered to the medium in concentrations from 0.5 – 2 µM for (30 and) 60 min. The dimethyl sulfoxide (DMSO) control was matched to the highest concentration of TPA applied. The displayed data is representative of at least 2 technical replicates in a minimum of 2 biological repeats for all performed experiments. The data of these replicates and repeats are shown in Figure S7 with the designation of the corresponding figures in the main manuscript.

In addition, cell viability assays using the xCELLigence® real-time cell analysis dual plate (RTCA DP) device (OLS OMNI Life Science GmbH & Co KG; Bremen, Germany) were carried out. For impedance-based real-time cell index measurement, cells were grown on E-Plate 16 PET (Agilent Technologies, Inc.). The interval for measurement sweeps was set to 15 min. ~ 5000 cells suspended in a total volume of 150 µl of growth medium were seeded in each well. After 24 h siRNA against DUSP4, 6, the combination of both or SCR was added equivalent to the protocol described above. Afterward, we acquired the measurement for up to 72 h. Raw data were analyzed with the RTCA software (OLS OMNI Life Science GmbH & Co KG). The data were normalized to the timepoint of inhibitor addition.

### NanoString® and statistical methods

Total RNA was extracted from 4 samples from each group: empty vector (EV), normal-appearing tissue in *NRAS* injected mice (NT), and the matched tumorous tissue (T). The concentration was measured on a DeNovix® DS-11 FX + und NanoDrop® ND-1000 (DeNovix Inc., Wilmington, USA), and RIN^e^ values were obtained on a 200 TapeStation (Agilent, Santa Clara, USA). The 12 mouse RNA samples that were used for further analyses had RIN^e^ values between 7.1 and 9.9 and were adjusted to a concentration of 100 ng/5 µl. Gene expression analysis was conducted using the nCounter® Mouse PanCancer Pathways Panel, which comprises 770 genes from 13 cancer-associated canonical pathways (NanoString, Seattle, USA).

Total RNA from DUSP4 silencing experiments in AKT/NRAS and AKT Cre/NRAS was analyzed in 3 biological replicates each (SCR vs. DUSP4 siRNA) with the nCounter® Mouse PanCancer Pathways Panel. Following the manufacturer’s instructions, 100 ng of total RNA was hybridized overnight with the reporter and capture code set at 65 °C. Excess probes were washed off using a two-step magnetic bead-based purification on the nCounter Prep station (NanoString Technologies). Elution from the beads, immobilization on a cartridge, and subsequent alignment followed. Data were collected using the nCounter Analyzer (NanoString Technologies) at 555 fields of view (FOV) through epifluorescence microscopy and CCD image acquisition.

Data were analysed with the nSolver™ analysis software Version 4.0 (NanoString Technologies). We ascertained that all samples passed quality control in the software. The nCounter Advanced Analysis 2.0 plug-in (NanoString Technologies) was employed for further analysis and hypothesis generation. We selected automated normalization by choosing those genes that minimize the pairwise variation statistics. For visualization, unsupervised clustering was chosen to generate a heatmap based on the normalized data counts of individual mRNAs. Differential expression was displayed as a volcano plot with individual genes’ − log10 (p-value) and log2 fold change compared to the control group. For differential expression analyses, *p*-value adjustments with the Benjamini-Yekutieli method were selected.

The nSolver path view module was employed to display the undirected global significance score of the included Kyoto Encyclopaedia of Genes and Genomes (KEGG) gene as a heatmap [29]

### Histology and immunohistochemistry, Protein isolation and Western blotting, Nucleic acid extraction and quantitative reverse transcription real-time polymerase chain reaction (qRT-PCR)

Please refer to the supplementary methods section for an overview of these techniques.

## Results

### NRAS^G12V^ induces hepatocarcinogenesis with robust upregulation of canonical effectors

**Fig. 1:**
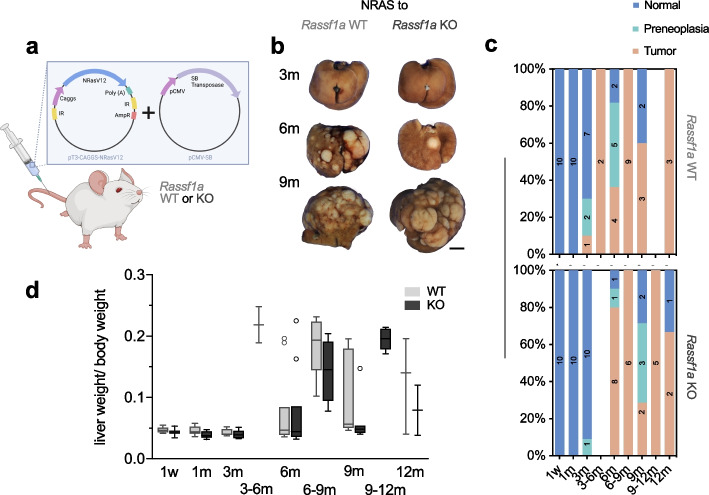
Hydrodynamic injection of *NRAS*^G12V^ in the mouse liver drives hepatocarcinogenesis (irrespective of *Rassf1a* genetic background). **a** Scheme of hydrodynamic injection into the tail vein with vector maps of the injected constructs. **b** Representative gross images of livers of *Rassf1a* WT mice injected with *NRAS*^G12V^ mutant forms at indicated time points. *Scale bar:* 0.5 cm. **c** Stacked bar charts visualizing the frequency of occurrence of neoplastic lesions *NRAS*^G12V^ injected *Rassf1a* WT and KO mice. **d** Comparison of the ratio of liver weight and body weight as surrogate for the tumour burden in *NRAS*^G12V^ injection groups sorted by *Rassf1a* background

**Fig. 2:**
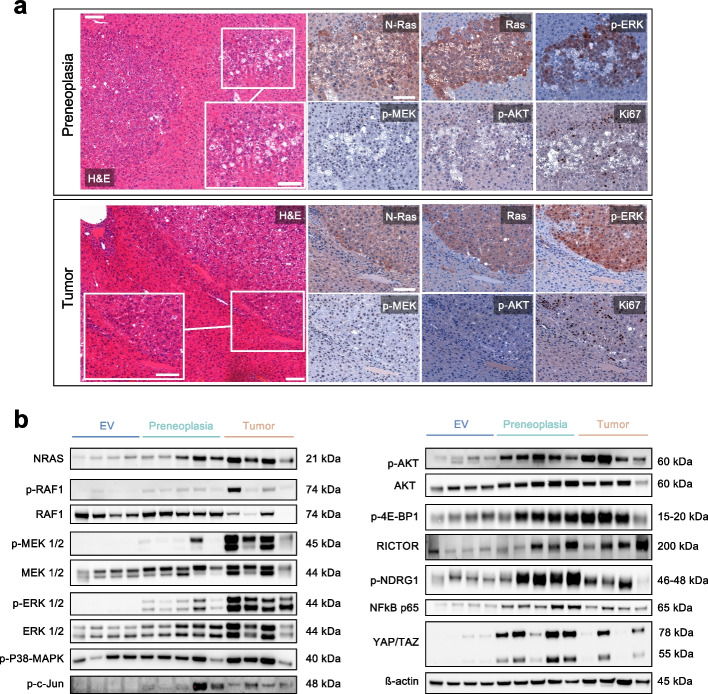
Histology and functional characterisation of *NRAS*^G12V^ dependent preneoplastic lesions and tumours. **a** Representative histological images two preneoplastic lesions (upper panel) and one tumour (lower panel) resulting from *NRAS*^G12V^ injections in *Rassf1a* WT mice. *Scale bar:* 100 µm / inset 50 µm. **b** Cropped Western blot analysis (full image of cut membrane that was hybridized with respective antibody in Figure S7) showing effective upregulation of NRAS and its effectors as well as the AKT/mTOR and Hippo pathways with increasing intensity in liver tissue containing preneoplastic lesions and tumours induced by *NRAS*^G12V^ injections as compared to empty vector (EV). Detailed interpretation: enhanced p-ERK cytoplasmic and nuclear positivity in comparison with the surrounding tissue; mitogen-activated protein kinase (MAPK) family member p38-MAPK with increased activation (i.e., phosphorylation) in the tumours, a potential explanation for the nuclear factor kappa B enhancer binding protein (NFκB) expression [30]; p–c-Jun more prominent in tumours, a possible effect of ERK activation that can stabilize c-Jun and increase c-Jun transcription through cAMP responsive element binding protein 1 (CREB1) and glycogen synthase kinase 3 (GSK3) [31]; p-AKT slightly upregulated by immunohistochemistry and Western blot analysis, while the corresponding levels of total AKT are increased. Note that p-S6rp and p-4EBP1 as downstream effectors of the PI3K-AKT-mTOR pathways were not apparently increased by immunohistochemistry (Figure S2). Yes1-associated transcriptional regulator (YAP) and transcriptional coactivator with PDZ-binding motif (TAZ) are elevated on the protein level. The major regulators of de novo lipogenesis Fatty acid synthase (FASN) and Stearoyl-CoA 9-desaturase 1 (SCD1) are upregulated

Upon HTVI of *NRAS*^G12V^, macroscopic hepatic tumours were detected as early as 3 months after the injection in the *Rassf1a* wildtype (WT) group, while the first grossly visible tumours were detected at the 6 months time point in *Rassf1a* knockout (KO) mice. Overall, 22 out of 60 (36.7%) mice developed grossly discernible tumours in the WT group, similar to 23 out of 62 (37.1%) mice in the KO group (Fig. 1b). Histologically, preneoplastic lesions (see definition below) were detectable in 7 WT mice (11.7%) and 5 KO mice (8.1%). Evaluation of the incidence per time point also yielded similar results between WT and KO, as inferred from Fig. 1c. Several mice required euthanasia before the predetermined time point and are represented in the intervals 3–6, 6–9, and 9–12 months. The liver weight/ body weight ratio was used as a surrogate parameter for tumour burden; again, no apparent differences between KO and WT mice were observed (Fig. 1d). Overall, tumour latency was 3–6 months, after which most mice displayed macroscopic tumours. As reported previously, the control group, which comprised untreated, 1xPBS, and EV injected mice did not develop tumours in the WT group, while 1 tumour each was detectable at 9 and 18 months in the *Rassf1a* KO cohort [19]. Histologically, the *NRAS*-induced tumours mostly had a well-circumscribed margin with a solid (partly also pseudocystic) growth pattern. They comprised cells reminiscent of hepatocytes with limited to moderate cytological atypia. Nuclei were hyperchromatic, and the ratio of the nuclear-cytoplasmic ratio was increased. Occasionally, vacuolated tumour areas could be observed, which is indicative of lipid accumulation inside the cells, while being much less pronounced when compared to our phosphatidylinositol-4,5-bisphosphate 3-kinase catalytic subunit alpha (*PIK3CA*) injection model [19]. Often tumour cells had amorphous eosinophilic inclusion bodies. Some tumours also showed intravascular growth, although we did not detect metastases outside the liver by macroscopic and microscopic evaluation during the autopsy. A subset of tumours possessed a central fibrotic core, populated by a distinct tumour cell population growing in strands or a ductular to cribriform pattern. These tumour cells were weakly positive for cytokeratin 7, thus imparting a biliary phenotype, while showing reduced expression of CPS1, a hepatocyte-specific marker (Figure S1). Apart from tumours, we defined the category of preneoplastic lesions. This distinction was based on a morphological comparison: tumours displayed the occasional presence of necrosis, expansive growth with evident compression or more diffuse infiltration of surrounding tissue, and macroscopic correlation as well as more overt cytologic signs of malignancy compared with preneoplastic lesions. The size of preneoplastic lesions was defined as more than 0.2 and less than 0.5 cm. Examples are shown in Fig. 2a. *Rassf1a* WT and KO tumours were indistinguishable at the histological level. As discussed above, the observation that *NRAS*^G12V^ injections alone led to tumorigenesis was deemed highly interesting, since the previous assumption had been that it would elicit senescence and consecutive clearance of early preneoplastic foci by the immune system [12]. The resulting tumours offered the possibility to study exclusively *RAS*-dependent hepatic tumorigenesis, so we continued with an in-depth analysis of *NRAS*^G12V−^induced tumours in *Rassf1a* WT mice.

On immunohistochemical (Fig. 2a) and Western blot evaluation (Fig. 2b), both tumours and preneoplastic lesions shared a common profile. An overexpression of pan-RAS and NRAS could be confirmed, which indicates the incorporation of the *NRAS*^G12V^ transgene. The immediate effector p-RAF1 was consequently increased at the protein level. In addition, augmented p-MEK positivity was hinted in the nuclei, while being more evident in the Western blot. Moreover, enhanced p-ERK cytoplasmic and nuclear positivity was evident compared to the surrounding tissue. Ki67 positive nuclei appeared to be more frequent in tumours when compared to preneoplastic lesions (Fig. 2a).

Altogether, through this comprehensive characterization, we were able to underline the strong upregulation of major known canonical effectors of RAS. Nonetheless, signalling via the PI3K-mTOR and Hippo pathways was also observed (detailed in the figure legend), suggesting a considerable level of crosstalk [32, 33]. Thus, despite being exclusively Ras-triggered, our model outlines the complexity of effects on oncogenic signalling via various effectors.

It should be noted, that the distinction between preneoplastic lesions and tumours was perfomed exclusively on the above-mentioned morphological criteria. At the molecular level, oncogenic pathways were gradually activated. Thus, in this model, we could not demonstrate a stepwise process of carcinogenesis. On these grounds, using the terms "early and late tumours" would also be justifiable as a replacement for preneoplasia and tumour.

### Identification of regulated genes in *NRAS*-driven hepatocarcinogenesis

To deepen our understanding of the process of *Ras*-dependent carcinogenesis, we performed NanoString® gene expression analyses using the nCounter® Mouse PanCancer Pathways Panel on total RNA extracted from tumour samples and corresponding non-tumorous samples (which, by histological assessment, contained preneoplastic lesions) from 4 mice (time point post-injection 3–9 months). As a control group of non-neoplastic liver tissue, samples from mice with normal histology injected with empty vector were chosen (time point post-injection 3–6 months). As expected, an upregulation of the Ras and MAPK pathway was observed. Also, the PI3K signalling was gradually increased from preneoplastic lesions to tumours.

**Fig. 3:**
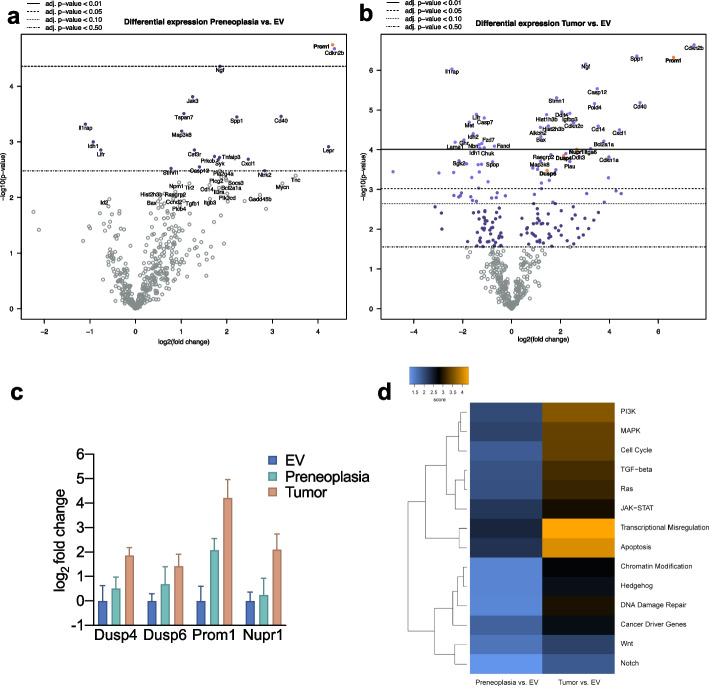
Transcriptional assessment of *NRAS*^G12V^ induced neoplastic lesions and identification of the novel effectors *Dusp4*, *Dusp6* and *Prom1* (CD133) (**a**) Volcano plot of differentially expressed genes obtained from NanoString® mRNA measurement using the mouse Pan Cancer pathway panel® in preneoplastic lesions and (**b**) tumours compared to empty vector. Genes that were analysed further due to rational selection are highlighted in yellow. **c** Differential expression of indicated genes from NanoString® analysis displayed as mean with 95 percent confidence interval. **d** Heatmap showing pathway scores obtained from the nSolver® software compared to empty vector. Orange indicates high pathway scores; blue indicates low scores (with undetermined/arbitrary functional dimension). The pathway scores are derived from KEGG, developed by Kanehisa Laboratories [34–36]

Evaluation of the pathway scores based on the Kyoto Encyclopedia of Genes and Genomes (KEGG) gene sets showed a progressive increase in the activity of several major cancer-related pathways when comparing preneoplastic tissue and tumour tissue against normal liver tissue. Transcriptional Misregulation and Apoptosis were the most prominently altered pathways (Fig. 3d).

**Fig. 4:**
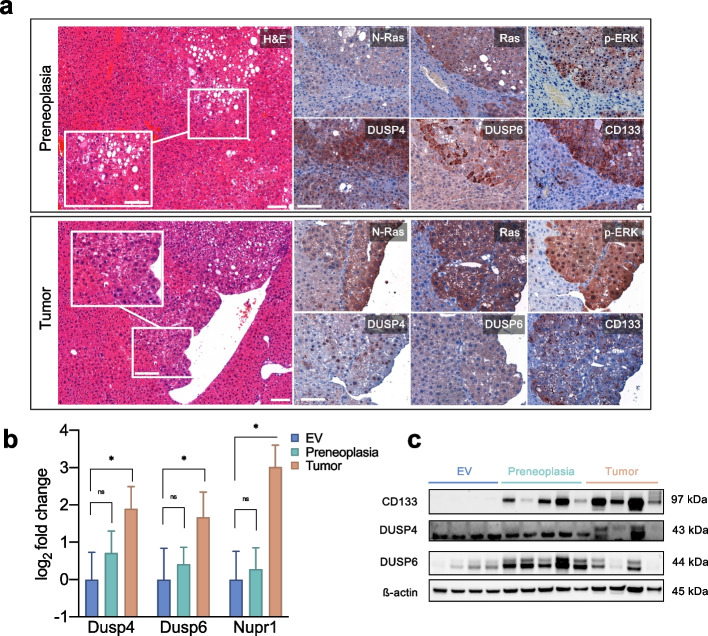
Validation of the selected target proteins by immunohistochemistry, RT-qPCR and Western Blot (**a**) Immunohistochemical analyses of *NRAS*^G12V^ -induced preneoplastic lesions (upper panels) and tumours (lower panels). DUSP4 overexpression in preneoplastic lesions can sometimes show cytoplasmic staining, while in tumours it is clearly enhanced in the nuclei. DUSP6 immunohistochemistry is more pronounced in preneoplastic lesions and hinted in tumours. CD133 shows a strong specific membranous staining already in preneoplastic lesions, which is maintained in tumours. *Scale bar:* 100 µm / inset 50 µm. **b** Quantitative real-time PCR analyses of selected targets. Kruskal–Wallis test for multiple comparisons. Identical RNA lysates as for the NanoString® analysis. Asterisk indicates *p*-value < 0.05; ns, not significant. **c** Cropped Western blots of the respective tumours and EV controls (full image of cut membrane that was hybridized with respective antibody in Figure S7). Molecular weights of observed bands are marked on the right

The differentially expressed genes analysis of preneoplastic lesions versus normal tissue only yielded 3 genes with an adjusted *p*-value less than or equal to 0.05, including *Prom1*, *Cdkn2b*, and *Ngf*. In contrast, the comparison of EV-injected samples with tumours detected 59 significantly differentially expressed genes with an adjusted *p*-value less than or equal to 0.05. Of note, among the most highly overexpressed genes, we again detected *Prom1*, *Cdkn2b*, and *Ngf* (Fig. 3a and 3b, Supplementary Data 1), which implies an early and sustained contribution throughout the process of carcinogenesis. The marked upregulation of *Cdkn2b*, while *Cdkn1a* and *Cdkn1c* were found elevated (Supplementary Data 1), strongly suggested an active oncogene senescence pathway, which was potentially counter-regulated by enhanced protein levels of CyclinD1 evident by immunohistochemistry (Figure S2). Presumably, the net effect of the combined changes was an evasion of the senescence mechanism with the development of preneoplastic lesions and tumours. The upregulation of *Ngf* argues for the presence of a positive feedback loop, since *Ngf* can cause sustained ERK1/2 activation and nuclear translocation [37]. Finally, the strong and early upregulation of *Prom1* (encoding CD133), which was also confirmed by immunohistochemistry (specific membranous expression), RT-qPCR, and Western blot (Fig. 4), was of particular interest. While CD133 is one of the most established markers for detecting and isolating cancer stem cells, including liver cancer, its function remains enigmatic [38]. For instance, it has been connected to oncogenic β-catenin and JAK/STAT pathways [39, 40]. Moreover, it has been purported that CD133 activity is enhanced by NFκB, which is also upregulated in our model [41]. Nonetheless, the exact interrelation with the RAS/MAPK pathway has not been reported, so we analysed this interaction further in the subsequent in vitro experiments.

Besides these genes, we also observed an upregulation of DUSP4 and 6 in comparing tumour samples and normal tissue (Fig. 3c). This upregulation is a compelling finding, since a digenic dependence on DUSP4 and DUSP6 has recently been reported, representing a potential therapeutic target [20]. While both DUSP4 and DUSP6 represent negative feedback regulators of ERK [42], they possess different substrate specificities. DUSP6 is a cytoplasmic ERK-specific phosphatase, and DUSP4 can additionally dephosphorylate JNK/p38 kinases [43]. Indeed, by immunohistochemistry, we could confirm an enhanced cytoplasmic positivity for DUSP6 in *NRAS*-induced tumours (Fig. 4a). At the same time, DUSP4 reacted predominantly in the nuclei (Fig. 4a* lower panel*), but in part also aberrantly in the cytoplasm of tumours (Fig. 4a* upper panel*) as it has been reported previously [44]. Upregulation in tumours was confirmed by RT-qPCR (Fig. 4b). Western blots displayed stronger bands in 2 out of 4 tumour samples, which were also evident in preneoplastic lesions in the case of DUSP6 (Fig. 4c). Finally, we confirmed the upregulation of *Nupr1* in tumours by RT-qPCR, a stress-induced protein involved in sorafenib resistance and NFκB and ERK pathway crosstalk [45]. DUSP4 and DUSP6, as well as CD133, were subsequently investigated more in-depth.

### Effects of modulations of the RAS/MEK/ERK pathway on DUSP4/6 and CD133 protein levels

**Fig. 5:**
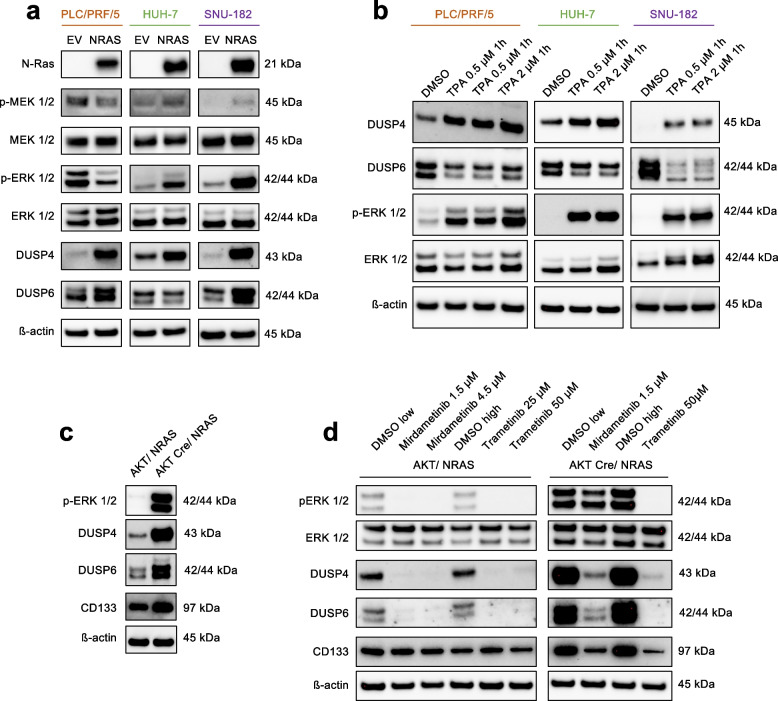
DUSP4, DUSP6, and CD133 are regulated by the RAS-MEK-ERK pathway in human and mouse liver cancer cell lines (**a**) *NRAS*^G12V^ transfections of human HCC cell lines with differential effects on pERK levels, while displaying a homogenous upregulation of DUSP4 and DUSP6. **b** Alternative activation of ERK via PKC using the phorbol ester TPA in the respective cell lines. **c** Baseline comparison of the mouse HCC cell lines AKT/NRAS and AKT Cre/NRAS demonstrating that the unique dependency on DUSP4 and DUSP6 overexpression is lost when the AKT pathway is additionally active. **d** Effects of MEK-inhibitors on selected target proteins in the AKT/NRAS and AKT Cre/NRAS cell lines. For all panels cropped Western Blots are shown. Full images of the cut membranes that were hybridized with the respective antibodies in Figure S7

Next, we set out to verify the regulation of the proteins mentioned above in human HCC cell lines (Figure S3). Six of 12 cell lines had detectable DUSP4 protein levels with weak expression in 3 cell lines (HUH7, HLE, and SNU-387), moderate expression in one cell line (PLC/PRF/5), and very high basal expression in 2 cell lines (MHCC97-L and SNU-449). Of note, the amount of DUSP4 protein did not correlate with pERK/ERK ratios. DUSP6 expression was detectable in 11 out of 12 HCC cell lines. All cell lines were positive for CD133, while some were more strongly positive than others. For the consecutive NRAS overexpression experiments, we used PLC/PRF/5, HUH-7, and SNU-182 with absent to moderate DUSP4 baseline expression. Transfection of these cell lines with pT3-CAGGS-NRasV12 led to a strong upregulation of NRAS protein expression (Fig. 5a). In response, the two cell lines, HUH-7 and SNU-182, exhibited an increase in the canonical effectors p-MEK1/2 and pERK1/2. Surprisingly, however, PLC/PRF/5 experienced a reduction in both p-MEK1/2 and p-ERK1/2. DUSP4 showed a strongly enhanced signal in all cell lines, and DUSP6 in PLC/PRF/5 and SNU-182. Apparently, additional NRAS^G12V^ transfection represented such a strong stimulus in PLC/PRF/5 cells that a negative feedback overcompensation occurred, likely mediated by DUSP4 and DUSP6. Interestingly, such overcompensation was not observed in the other two cell lines, suggesting that a threshold of “oncogene overdose” is required to reach the tipping point.

To investigate the specificity of these findings to NRAS activation, we subjected the cell lines to another method of ERK hyperactivation by treatment with the phorbol ester TPA, which induces pERK1/2 via protein kinase C (PKC) [28]. Strong activation of ERK1/2 was detected in all cell lines (Fig. 5b), and protein levels of DUSP4 were strongly upregulated in a dose-dependent manner. In contrast to NRAS-mediated ERK-activation, protein levels of DUSP6 consistently decreased. Compellingly, in this experiment, no overcompensation was evident in PLC/PRF/5 cells, which indicates that this phenomenon could be specific to the RAS/MEK/ERK cascade triggered directly by strong oncogenic signalling through *NRAS*^G12V^. Moreover, this phenomenon could also rely on increased DUSP6 expression, which was contrarily regulated in the TPA treatments.

In addition, we studied the effects of MEK inhibition on the selected target proteins. For this experiment, we employed the AKT/NRAS cell line, derived from a murine HCC, which developed after HTVI of myristoylated AKT^flox/flox^ and NRAS^G12V^ [27], and its derivative AKT Cre/NRAS. Here AKT had been removed by the Cre recombinase, leading to a pure NRAS addiction as opposed to the combined AKT and NRAS oncogene dependency in the original cell line. The baseline characteristics of AKT/NRAS and AKT Cre/NRAS regarding DUSP4, DUSP6, and CD133 were very distinct (Fig. 5c). Indeed, AKT Cre/NRAS displayed a much higher activation of ERK1/2 and correspondingly high protein levels of DUSP4 and DUSP6, while CD133 was slightly increased. This effect argues for an exclusive role of DUSP4 and DUSP6 in NRAS dependency, which is attenuated upon additional concurrent activation of AKT.

We treated these two cell lines with the MEK inhibitors mirdametinib and trametinib for 24 h (Fig. 5d). As a result, DUSP4 and DUSP6 protein levels were strongly diminished in both cell lines. Also, pERK1/2 levels were reduced as expected in all treatment groups (although the effect of mirdametinib on ERK phosphorylation was limited in AKT Cre/NRAS). CD133 levels decreased considerably upon MEK inhibition in the purely NRAS-addicted AKT Cre/NRAS cell line. This finding argues for a direct relation between RAS-signalling and CD133 expression levels, confirming the results obtained in the mouse model.

**Fig. 6:**
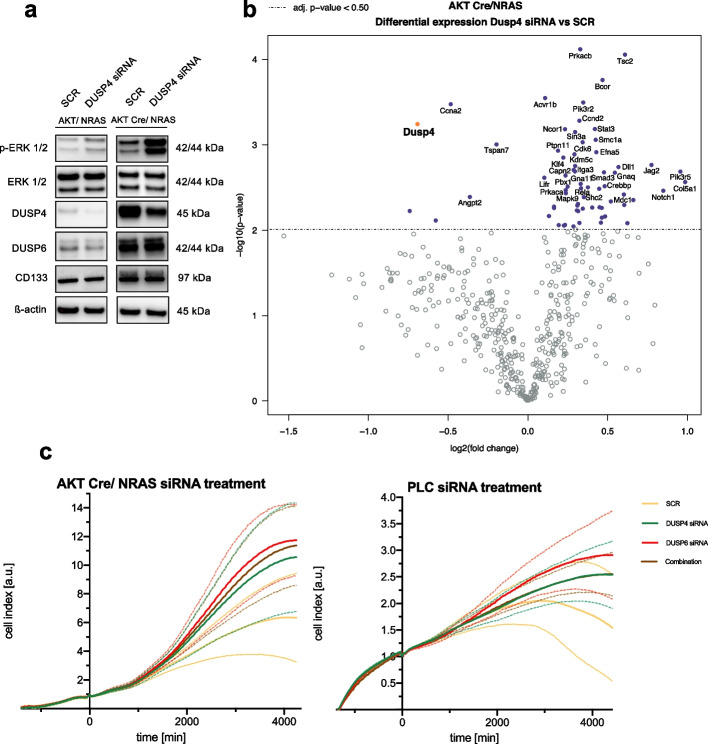
Effects of *Dusp4* silencing in *AKT/NRAS* and purely *NRAS* driven mouse HCC cell lines. **a** Western blot confirmation of *Dusp4* silencing in the mouse AKT/NRAS and AKT Cre/NRAS cell lines. Increased pERK1/2 levels and decreased DUSP4 protein levels are apparent (Cropped Western Blots; full image of the cut membrane that was hybridized with respective antibody in Figure S7). **b** Volcano plot of differentially expressed genes obtained from NanoString® mRNA measurement using the mouse Pan Cancer pathway panel® in AKT Cre/NRAS *Dusp4* silenced cells versus SCR negative control. *Dusp4* is highlighted in orange. **c** cCELLigence® proliferation measurements of AKT Cre/NRAS (left) and PLC (right). siRNA addition at time point 0. Data presented as mean (continuous line) with standard deviation (dashed line). Two repeats with 3 replicates each for AKT Cre/Nras, single experiment with 3 replicates in the case of PLC

Finally, we performed siRNA-mediated gene silencing of DUSP4 in these two murine cell lines. Moderate silencing efficiency was achieved on the protein level of DUSP4 and pERK1/2 (Fig. 6a). Downregulation of DUSP4 elicited an increase in pERK1/2 levels in both cell lines, especially in the AKT Cre/NRAS cell line. Protein levels of CD133 and DUSP6 were unaltered.

Subsequently, NanoString® gene expression analysis was conducted to identify differentially expressed genes following siRNA-mediated *Dusp4* silencing in the AKT Cre/NRAS cell line (Fig. 6b). Some markers of cell cycle progression, including *Ccnd2* and *Cdk6*, were upregulated. On the other hand, apoptosis-related genes were diminished, including *Casp12* and *Endog*, implying a pro-proliferative and anti-apoptotic effect mediated by *Dusp4* gene silencing. Furthermore, several genes related to the MAPK pathway were upregulated, including *Map3k5*, *Map3k*, *Mapk9,* and *Prkca* (except for downregulated *Map2k6*), which suggests a release of formerly active negative feedback regulation (Supplementary Data 1). Additionally, we compared the differentially expressed genes between AKT Cre/NRAS vs. AKT/NRAS (Figure S4, Supplementary Data 1). This comparison verified the reduction in *Akt1* expression. Moreover, we found a strong upregulation of *Cdkn2a* in the AKT Cre/NRAS cell line, which could imply an increased tendency towards senescence and cell cycle inhibition in this cell line. Again, the purely NRAS defined oncogene addiction appeared to be more prone to senescence response, which can be overcome by the additional activity of the AKT pathway and was mirrored by much slower growth characteristics of the AKT Cre/NRAS cell line.

Subsequently, we evaluated the effects of DUSP4 and DUSP6 silencing, alone and combined, in the HCC cell line PLC and the murine AKT Cre/NRAS cell line using an impedance-based real-time measurement (xCELLigence®, Fig. 6c). We detected an increased growth rate for DUSP4 and DUSP6 silencing and the combination of both compared to SCR. In a parallel measurement with adjusted parameters, we further validated the effectiveness of the silencing in both instances (Figure S5).

### *KRAS* mutations in CCA cell lines and human samples and NRAS mutations in CCA mouse models correlate with increased DUSP4 expression

**Fig. 7:**
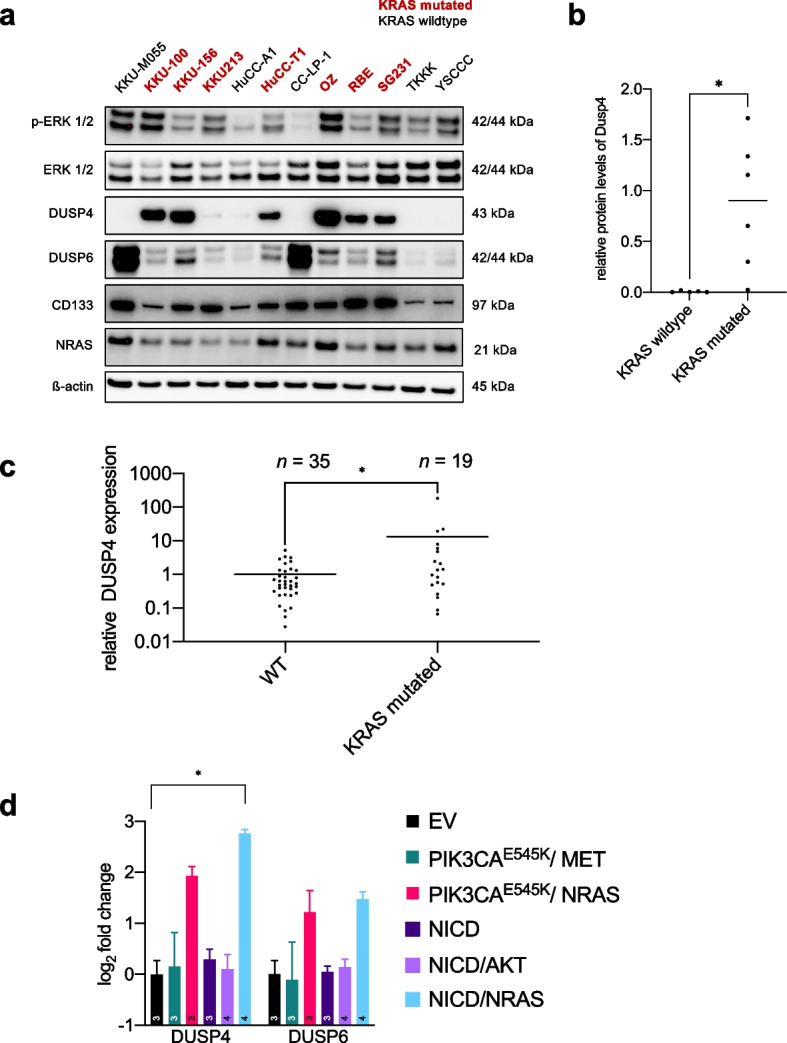
RAS-dependence of DUSP4 upregulation in human and murine CCA (**a**) Western blot analysis of human CCA cell lines showing varying DUSP4, DUSP6, and CD133 baseline levels (Cropped Western Blots; full image of the cut membrane that was hybridized with respective antibody in Figure S7). Cell lines with known *KRAS* mutations are highlighted. **b** Quantification of DUSP4 protein levels normalized to β-actin bands with respect to *KRAS* mutational status. Mann–Whitney test. Asterisk indicates *p*-value < 0.05. **c** Quantitative real-time PCR of DUSP4 expression in a local cohort of 54 CCA patients with and without KRAS mutation as determined by high-resolution melting analysis; KRAS 12–13 16x, KRAS 59–61 2x, KRAS 146 1x. Line equals mean value. Asterisk corresponds to *p*-value < 0.05 (Mann–Whitney-U-Test) (**d**) Quantitative real-time PCR analyses of DUSP4 and DUSP6 expression levels of tumours in different mouse models compared to normal liver tissue (empty vector, EV). The PIK3CA-based models led to the formation of HCC, and the NICD-based models induced the development of CCA. Asterisk signifies *p*-value < 0.05; all other comparisons were not significant (Kruskal–Wallis test with multiple comparisons). The number in bars equals the number of mice

Since in human HCC *RAS* mutations are rare, we resorted to CCA cell lines to verify the effect of *RAS* mutations on DUSP4 transcriptional levels. Therefore, we studied 12 CCA cell lines, of which we had previously confirmed the *KRAS* mutational status [4]. We found a significant increase (*p* = 0.043, Mann–Whitney test) of DUSP4 protein levels relative to β-actin in the 7 *KRAS* mutated cholangiocarcinoma cell lines when compared to *KRAS* wildtype CCA cell lines (Fig. 7).

In addition, we analysed our local CCA cohort, which had been screened before for mutations in *KRAS,* using a high-resolution melting curve analysis. From this cohort, we compared 35 *KRAS* WT to 19 *KRAS* mutated cases by qPCR (Fig. 7c). A statistically significant upregulation of DUSP4 expression levels in the KRAS-mutated tumours was detected (*p* = 0.048, Mann–Whitney test).

Furthermore, we confirmed the regulation of DUSP4 in response to NRAS^G12V^. For this purpose, we selected the CCA cell line KKU-M055, which does not harbour KRAS or NRAS mutations (Figure S6) and has a low DUSP4 baseline expression. Following the transfection of NRAS^G12V^, we observed a statistically significant (*p* = 0.026, Mann- Whitney-Test) upregulation of Dusp4 protein levels, while Dusp6 remained unaltered. Thus, the observation of Dusp4 overexpression under the influence of activated NRAS could be confirmed in a CCA cell, similar to the regulation in HCC cell lines described earlier.

Finally, we investigated further HCC and CCA mouse models to corroborate the dependency of DUSP expression on the injection of NRAS^G12V^ across entities. Specifically, we analysed PIK3CA^E545K^/ MET and PIK3CA^E545K^/ NRAS^G12V^, which induce HCC [46]. An increase of *Dusp4* and *Dusp6* transcripts was detected. Similarly, a statistically significant difference for Dusp4 expression was observed in the NICD/NRAS CCA model [47] compared to EV (adj *p* = 0.023, Kruskal–Wallis test, Dunn’s multiple comparisons). No statistically significant difference was observed in the NICD only and NICD/AKT models [48]. Dusp6 showed a trend of being elevated in the NRAS combined model. Thus, we could clearly define a dependency of Dusp4 upregulation based on the presence of activated NRAS. This observation was specific to NRAS and could not be mirrored by e.g., AKT activation.

## Discussion

In this study, we demonstrated that hepatocarcinogenesis can be triggered by the sole HTVI of only *NRAS*^G12V^, which opposes previously reported observations where *NRAS*^G12V^ was insufficient to elicit tumorigenesis [12]. In the mentioned study, pre-malignant hepatocytes were cleared by the immune system through a process defined by CD4^+^ T-cells and macrophages as a purported result of oncogene-induced senescence. One potential explanation for this discrepancy could be the difference in the mouse genetic backgrounds. While C57BL/6 × 129 Sv mice were used in our study, the other investigation had been conducted in a pure C57BL/6 background. Furthermore, in the C57BL/6 × 129 Sv background, we had previously reported a tendency to develop tumours by the HTVI of either *PIK3CA* E545K or H1047R alone, which had similarly conflicted with earlier reports, that these alterations would not elicit carcinogenesis in an FVB/N genetic background [46]. We found no synergism between *Rassf1a* knockout and *NRAS*, which strengthens our previously reported lack of cooperativity between *Rassf1a* and *PIK3CA* mutant forms [19]. Focusing on wildtype animals, the occurrence of tumours following *NRAS* injections alone argues for a comparatively high tumour susceptibility in the mixed C57BL/6 × 129 Sv background, although in the literature, the parental strain 129 Sv is described to feature an overall low tumour incidence [49].

Ultimately, this increased susceptibility provided us with the unique possibility to study the molecular details of a purely *NRAS*-defined mode of hepatocarcinogenesis. This is of high interest since it is known that human HCC depends heavily on the activation of the RAS/MEK/ERK signalling cascade [6]. Even though the frequency of *NRAS* and *KRAS* mutations combined is relatively low at just under 3%, many more indirect mechanisms exist that lead to a similar net effect on the activity of this pathway, such as the aberrant activation of upstream growth factors and the increase of positive or decrease negative regulators [50]. Thus, developing models that allow the precise study of RAS/MEK/ERK pathway deregulation in HCC is paramount. Accordingly, in our proposed mouse model, we could determine a strong upregulation of the canonical effectors of RAS signalling, while confirming a robust upregulation of non-canonical or indirect RAS effectors such as p38-MAPK with subsequent NFκB and p–c-Jun. Nonetheless, we could not achieve a complete specificity for the Ras pathway, since the signalling was also dispersed to other oncogenic pathways, particularly to AKT-mTOR signalling, which was mirrored by an increase in RICTOR and targets of mTORC1. Furthermore, we found an upregulation of the Hippo pathway. Thus, a significant crosstalk to other oncogenic pathways occurred, which could pose significant challenges to targeting RAS effectors under active upstream signalling. Indeed, therapeutic concepts have been proposed, for example, for *KRAS* mutant pancreatic cancer, in which the combined inhibition of mTORC1/2 and MEK precludes adaptive resistance [51].

In a more detailed gene expression analysis, we identified several significantly deregulated genes, including *Dusp4*, *Dusp6,* and *Prom1*/CD133. The abundance of upregulated members of the DUSP family led us to further study their association with RAS signalling in HCC. Their concurrent upregulation was particularly interesting, since a recent report had suggested a digenic dependence on DUSP4 and DUSP6 in MAPK-driven cancers [20].

Initially, we confirmed that high expression of DUSP4 and DUSP6 was indeed a feature of several human HCC cell lines, which argues for the importance of these proteins even in the absence of direct *RAS* mutations. Experimentally, we could further demonstrate that DUSP4 and DUSP6 upregulation was consistently detected following *NRAS*^G12V^ transfection. More specifically, by using the phorbol ester TPA, we could deduce that ERK activation was required for the observed increase in DUSP4 protein levels, while DUSP6 experienced an inverse regulation by mere ERK activation. Thus, the positive influence on DUSP6 should depend on factors upstream of ERK or other targets unique to RAS activation. One intriguing finding of these experiments was that NRAS transfection led to a paradoxical decrease of pERK levels in the cell line PLC/PRF/5. This observation hints at an overshooting feedback inhibition that can result from robust stimulation of RAS. A similar mechanism for DUSP4 and DUSP6 upregulation could be envisioned in our mouse model of *NRAS*-induced hepatocarcinogenesis. Indeed, the extreme upregulation of the RAS/ERK pathway could be tempered by DUSP4 and DUSP6 as rheostats, thus potentially favouring evasion of oncogene-induced senescence. Interestingly, when we investigated a purely *NRAS*-dependent murine cancer cell line, we observed a much higher baseline level of DUSP4 and DUSP6 compared to its dual NRAS and AKT-addicted counterpart, which is suggestive of a certain exclusivity towards the RAS oncogenic pathway.

Whether DUSP4 and DUSP6 serve as oncogenes or tumour suppressors is still under debate [52]. Silencing of DUSP4 resulted in a pro-proliferative and anti-apoptotic effect in the AKT Cre/NRAS cell line, which was mirrored by an increased growth rate in an impedance-based growth assay. To put this finding into perspective, it has been described that DUSP4 can have opposite effects on proliferation. One paper reported DUSP4 deficiency leading to impaired cell proliferation and cell death in NRAS and BRAF mutant melanoma cells. In contrast, in non-melanoma cell lines with BRAF mutation, DUSP4 silencing did not affect their growth (a glioma cell line and a colorectal cancer cell line) [53]. Moreover, DUSP4 loss increased invasiveness in pancreatic cancer, and restoration of DUSP4 expression reversed this effect [54]. Finally, in colorectal cancer models, silencing of DUSP4 enhanced cell proliferation and invasiveness, most reminiscent of the effect we observed in HCC cell lines [55]. Therefore, higher levels of DUSP4 would rather be suggestive of a function as a tumour suppressor. However, at the same time, it might be envisioned that DUSP4 and DUSP6 maintain pERK at tolerable levels allowing the tumour cells to reach a state of stemness to overcome proliferative insufficiency. Altogether, a definite conclusion for a therapeutic concept targeting DUSP4 cannot be reached here. However, we add another layer of complexity, serving as a note of caution when studying DUSP-targeting therapeutics. The dependency on mutational and cancer entities should be controlled.

Finally, NRAS overactivation was also defined by strong CD133 cancer stem cell antigen upregulation, already present in early tumours. In vitro, we described a hitherto unknown regulation of CD133 protein levels by the RAS pathway activity. This stemness player could also contribute to counteracting oncogene-induced senescence mechanisms.

While *NRAS* mutations rarely occur in human HCC, they are more frequent in CCA, another primary liver tumour, where we confirmed the dependence of DUSP4 protein expression on the *KRAS* mutational status in cell lines and patient samples. This finding hints at a conserved mechanism active across different cancer subtypes.

## Conclusions

Opposite to earlier assumptions, our study demonstrates that *NRAS*^G12V^ is sufficient to drive hepatocarcinogenesis in a mouse HTVI model. Further characterisation of this model defined the upregulation of the cancer stem cell antigen CD133 and the dual specificity phosphatases DUSP4 and DUSP6 as defining features of *RAS*-induced HCCs. By serving as a rheostat to RAS pathway activity, these proteins can offer a survival benefit to the cancer cells and putatively allow for evasion of oncogene-induced senescence. Given that CCA also shows DUSP4 upregulation in the presence of *RAS* mutations, we assume our observations can be extended to other cancer entities.

### Supplementary Information

**Additional file 1: Supplementary data 1.** NanoString® differentially expressed genes with indicated cut-offs.

**Additional file 2: **Supplementary Figures.

**Additional file 3: **Supplementary Tables.

**Additional file 4: **Supplementary Methods.

## Data Availability

The datasets supporting the conclusions of this article are included within the article and its additional file. Moreover, the gene expression microarray data that support the findings of this study are openly available in NCBI's Gene Expression Omnibus and are accessible through https://www.ncbi.nlm.nih.gov/geo/query/acc.cgi?acc=GSE216200, GEO Series accession number GSE216200 (mouse liver lysate comparisons) and https://www.ncbi.nlm.nih.gov/geo/query/acc.cgi?acc=GSE217233, GEO Series accession number GSE217233 (DUSP4 silencing comparisons).

## Abbreviations

iCCA: Intrahepatic cholangiocarcinoma
CREB1: CAMP responsive element binding protein 1
DMSO: Dimethyl sulfoxide
DUSP4: Dual Specificity Phosphatase 4
DUSP6: Dual Specificity Phosphatase 6
EV: Empty vector
FASN: Fatty acid synthase
GSK3: Glycogen synthase kinase 3
HCC: Hepatocellular carcinoma
HGF: Hepatocyte growth factor
HTVI: Hydrodynamic tail vein injection
IHC: Immunohistochemistry
KEGG: Kyoto Encyclopedia of Genes and Genomes
KO: Rassf1a knockout
KRAS: Kirsten rat sarcoma virus
MAPK: Mitogen-activated protein kinase
MEK: Mitogen-activated protein kinase kinase
NFκB: Kappa B enhancer binding protein
NRAS: Neuroblastoma RAS viral oncogene homolog
NT: Normal appearing tissue in NRAS injected mice
PBS: Phosphate-buffered saline
pERK: Phosphorylated extracellular-signal regulated kinase
PIK3CA: Phosphatidylinositol-4,5-bisphosphate 3-kinase catalytic subunit alpha
PKC: Protein kinase C
qRT-PCR: Quantitative reverse transcription real-time polymerase chain reaction
RAF: Rapidly accelerated fibrosarcoma
SCD1: Stearoyl-CoA 9-desaturase 1
T: Tumorous tissue
TPA: 12-O-Tetradecanoylphorbol-13-acetate
TrkA: Tropomyosin receptor kinase A
WT: Rassf1a wildtype
YAP: Yes1 associated transcriptional regulator

## References

[1] Sung H, Ferlay J, Siegel RL, Laversanne M, Soerjomataram I, Jemal A (2021). Global Cancer Statistics 2020: GLOBOCAN Estimates of Incidence and Mortality Worldwide for 36 Cancers in 185 Countries. CA Cancer J Clin.

[2] Banales JM, Cardinale V, Carpino G, Marzioni M, Andersen JB, Invernizzi P (2016). Expert consensus document: Cholangiocarcinoma: current knowledge and future perspectives consensus statement from the European Network for the Study of Cholangiocarcinoma (ENS-CCA). Nat Rev Gastroenterol Hepatol.

[3] Kanwal F, Singal AG (2019). Surveillance for Hepatocellular Carcinoma: Current Best Practice and Future Direction. Gastroenterology.

[4] Scheiter A, Hierl F, Winkel I, Keil F, Klier-Richter M, Coulouarn C, et al. Wnt/beta-Catenin-Pathway Alterations and Homologous Recombination Deficiency in Cholangiocarcinoma Cell Lines and Clinical Samples: Towards Specific Vulnerabilities. J Pers Med. 2022;12. 10.3390/jpm12081270 PMC9410222 36013219

[5] Moon Simon Weonsang HR. MAPK/ERK Signaling Pathway in Hepatocellular Carcinoma. Cancers (Basel). 2021;13:3026-NA. 10.3390/cancers13123026 PMC8234271 34204242

[6] Li Guo‑Dong; Shi, Zhe; Qi, Li‑Li; Zhou, Li‑Yuan; Fu, Ze‑Xian LZ. The Ras/Raf/MEK/ERK signaling pathway and its role in the occurrence and development of HCC (Review). Oncol Lett. 2016;12:3045–50. 10.3892/ol.2016.5110 PMC5103898 27899961

[7] Hoffmann Lin; Xiao, Zhi; Longerich, Thomas; Büchler, Markus W.; Schemmer, Peter KS. Correlation of gene expression of ATP-binding cassette protein and tyrosine kinase signaling pathway in patients with hepatocellular carcinoma. Anticancer Res. 2011;31:3883–90. 22110214

[8] Chen Yan; Jiang, C.-Y.; Wei, L.-X.; Wang, Yanrong; Dai, G.-H. LS. Expression and prognostic role of pan-Ras, Raf-1, pMEK1 and pERK1/2 in patients with hepatocellular carcinoma. Eur J Surg Oncol. 2011;37:513–20. 10.1016/j.ejso.2011.01.023 21324414

[9] Zhang Hao; Han, Feng; Shao, Xiaowen; Liu, Yun; Ma, Xuda; Wang, Zun; Qiang, Zhaoyan; Li, Yongmei XZ. Sp1‐regulated transcription of RasGRP1 promotes hepatocellular carcinoma (HCC) proliferation. Liver Int. 2018;38:2006–17. 10.1111/liv.13757 29655291

[10] Sirivatanauksorn Vorapan; Srisawat, Chatchawan; Khongmanee, Amnart; Tongkham, Chalita YS. Differential expression of sprouty genes in hepatocellular carcinoma. J Surg Oncol. 2011;105:273–6. 10.1002/jso.22095 21932411

[11] Wang Benchen; Lou, Jiamin; Li, Jianhao; Liu, Zhenguo; Li, Ang; Cui, Guangying; Ren, Zhigang; Yu, Zujiang HR. The Function of the HGF/c-Met Axis in Hepatocellular Carcinoma. Front cell Dev Biol. 2020;8 NA:55-NA. 10.3389/fcell.2020.00055 PMC7018668 32117981

[12] Kang TW, Yevsa T, Woller N, Hoenicke L, Wuestefeld T, Dauch D (2011). Senescence surveillance of pre-malignant hepatocytes limits liver cancer development. Nature.

[13] Ho Chunmei; Mattu, Sandra; Destefanis, Giulia; Ladu, Sara; Delogu, Salvatore; Armbruster, Julia; Fan, Lingling; Lee, Susie A.; Jiang, Lijie; Dombrowski, Frank; Evert, Matthias; Chen, Xin; Calvisi, Diego F. CW. AKT (v-akt murine thymoma viral oncogene homolog 1) and N-Ras (neuroblastoma ras viral oncogene homolog) coactivation in the mouse liver promotes rapid carcinogenesis by way of mTOR (mammalian target of rapamycin complex 1), FOXM1 (forkhead box M1)/SKP2, . Hepatology. 2011;55:833–45. 10.1002/hep.24736 PMC3269553 21993994

[14] Ju Sang Hoon; Kim, Young; Baek, Sinhwa; Chung, Sook In; Seong, Jinsil; Han, Kwang Hyub; Ro, Simon Weonsang HLA. Investigation of Oncogenic Cooperation in Simple Liver-Specific Transgenic Mouse Models Using Noninvasive In Vivo Imaging. PLoS One. 2013;8:e59869-NA. 10.1371/journal.pone.0059869 PMC3610734 23555816

[15] Xu Susie A.; Ho, Coral; Bommi, Prashant V.; Huang, Shiang; Cheung, Siu Tim; Dimri, Goberdhan P.; Chen, Xin CL. Bmi1 Functions as an Oncogene Independent of Ink4A/Arf Repression in Hepatic Carcinogenesis. Mol Cancer Res. 2009;7:1937–45. 10.1158/1541-7786.MCR-09-0333 PMC2796287 19934271

[16] Xin B, Yamamoto M, Fujii K, Ooshio T, Chen X, Okada Y (2017). Critical role of Myc activation in mouse hepatocarcinogenesis induced by the activation of AKT and RAS pathways. Oncogene.

[17] Dubois Emmanuel; Zalcman, Gérard; Levallet, Guénaëlle FB. RASSF1A, puppeteer of cellular homeostasis, fights tumorigenesis, and metastasis-an updated review. Cell Death Dis. 2019;10:928. 10.1038/s41419-019-2169-x PMC6895193 31804463

[18] Schagdarsurengin Ludwig; Steinemann, Doris; Flemming, Peer; Kreipe, Hans; Pfeifer, Gerd P.; Schlegelberger, Brigitte; Dammann, Reinhard UW. Frequent epigenetic inactivation of the RASSF1A gene in hepatocellular carcinoma. Oncogene. 2003;22:1866–71. 10.1038/sj.onc.1206338 12660822

[19] Scheiter A, Evert K, Reibenspies L, Cigliano A, Annweiler K, Müller K (2022). RASSF1A independence and early galectin-1 upregulation in PIK3CA-induced hepatocarcinogenesis: new therapeutic venues. Mol Oncol.

[20] Ito T, Young MJ, Li R, Jain S, Wernitznig A, Krill-Burger JM (2021). Paralog knockout profiling identifies DUSP4 and DUSP6 as a digenic dependence in MAPK pathway-driven cancers. Nat Genet.

[21] Lake Sônia A. L.; Müller, Jürgen DC. Negative feedback regulation of the ERK1/2 MAPK pathway. Cell Mol Life Sci. 2016;73:4397–413. 10.1007/s00018-016-2297-8 PMC5075022 27342992

[22] Keyse SM (2000). Protein phosphatases and the regulation of mitogen-activated protein kinase signalling. Curr Opin Cell Biol.

[23] Woods David A.D.; Cherwinski, Holly; Bosch, E; Lees, Emma; McMahon, Martin DP. Raf-induced proliferation or cell cycle arrest is determined by the level of Raf activity with arrest mediated by p21Cip1. Mol Cell Biol. 1997;17:5598–611. 10.1128/mcb.17.9.5598 PMC232408 9271435

[24] Zhu Douglas; McMahon, Martin; Bishop, J. Michael JW. Senescence of human fibroblasts induced by oncogenic Raf. Genes Dev. 1998;12:2997–3007. 10.1101/gad.12.19.2997 PMC317194 9765202

[25] Kaneko K, Liang Y, Liu Q, Zhang S, Scheiter A, Song D, et al. Identification of CD133+ intercellsomes in intercellular communication to offset intracellular signal deficit. Elife. 2023;12:RP86824. 10.7554/eLife.86824 PMC10581692 37846866

[26] Carlson Joel L.; Kirchhof, Nicole; McIvor, R. Scott; Largaespada, David A. CM. F. Somatic integration of an oncogene-harboring Sleeping Beauty transposon models liver tumor development in the mouse. Proc Natl Acad Sci U S A. 2005;102:17059–64. 10.1073/pnas.0502974102 PMC1287966 16286660

[27] Ho C, Wang C, Mattu S, Destefanis G, Ladu S, Delogu S (2012). AKT (v-akt murine thymoma viral oncogene homolog 1) and N-Ras (neuroblastoma ras viral oncogene homolog) coactivation in the mouse liver promotes rapid carcinogenesis by way of mTOR (mammalian target of rapamycin complex 1), FOXM1 (forkhead box M1)/SKP2. Hepatology.

[28] Frost JA, Geppert TD, Cobb MH, Feramisco JR (1994). A requirement for extracellular signal-regulated kinase (ERK) function in the activation of AP-1 by Ha-Ras, phorbol 12-myristate 13-acetate, and serum. Proc Natl Acad Sci U S A.

[29] Kanehisa Miho; Tanabe, Mao; Sato, Yoko; Morishima, Kanae MF. KEGG: new perspectives on genomes, pathways, diseases and drugs. Nucleic Acids Res. 2016;45:D353–61. 10.1093/nar/gkw1092 PMC5210567 27899662

[30] Vlahopoulos SA (2017). Aberrant control of NF-κB in cancer permits transcriptional and phenotypic plasticity, to curtail dependence on host tissue: molecular mode. Cancer Biol Med.

[31] Lopez-Bergami P, Huang C, Goydos JS, Yip D, Bar-Eli M, Herlyn M (2007). Rewired ERK-JNK signaling pathways in melanoma. Cancer Cell.

[32] Mendoza MC, Er EE, Blenis J (2011). The Ras-ERK and PI3K-mTOR pathways: cross-talk and compensation. Trends Biochem Sci.

[33] Azad T, Rezaei R, Surendran A, Singaravelu R, Boulton S, Dave J, et al. Hippo Signaling Pathway as a Central Mediator of Receptors Tyrosine Kinases (RTKs) in Tumorigenesis. Cancers (Basel). 2020;12. 10.3390/cancers12082042 PMC7463967 32722184

[34] Kanehisa M, Goto S (2000). KEGG: Kyoto Encyclopedia of Genes and Genomes. Nucleic Acids Res.

[35] Kanehisa M (2019). Toward understanding the origin and evolution of cellular organisms. Protein Sci.

[36] Kanehisa M, Furumichi M, Sato Y, Kawashima M, Ishiguro-Watanabe M (2023). KEGG for taxonomy-based analysis of pathways and genomes. Nucleic Acids Res.

[37] Brightman DA, FA. F.  (2000). Differential feedback regulation of the MAPK cascade underlies the quantitative differences in EGF and NGF signalling in PC12 cells. FEBS Lett.

[38] Zhu Xiangfang; Yan, Mingxia; Yao, Ming; Ge, Chao; Gu, Jianren; Li, Jinjun ZH. Cancer stem/progenitor cells are highly enriched in CD133+CD44+ population in hepatocellular carcinoma. Int J cancer. 2010;126:2067–78. 10.1002/ijc.24868 19711346

[39] Mak Allison M.L.; Kittanakom, Saranya; Stewart, Jocelyn M.; Chen, Ginny I.; Curak, Jasna; Gingras, Anne-Claude; Mazitschek, Ralph; Neel, Benjamin G.; Stagljar, Igor; Moffat, Jason AB. N. Regulation of CD133 by HDAC6 Promotes β-Catenin Signaling to Suppress Cancer Cell Differentiation. Cell Rep. 2012;2:951–63. 10.1016/j.celrep.2012.09.016 PMC3590846 23084749

[40] Won Byung Hak; Yi, Eun Hee; Choi, Kyung Ju; Kim, Eun Kyung; Jeong, Jong Min; Lee, Jae Ho; Jang, Ja June; Yoon, Jung Hwan; Jeong, Won-Il; Park, In Chul; Kim, Tae Woo; Bae, Sun Sik; Factor, Valentina M.; Ma, Stephanie; Thorgeirsson, Snorri S.; Lee, Yun-Han CK. Signal transducer and activator of transcription 3-mediated CD133 up-regulation contributes to promotion of hepatocellular carcinoma. Hepatology. 2015;62:1160–73. 10.1002/hep.27968 PMC5049669 26154152

[41] Kang Suhyun; Ko, Jesang MSK. Roles of CD133 in microvesicle formation and oncoprotein trafficking in colon cancer. FASEB J. 2018;33:4248–60. 10.1096/fj.201802018R 30521383

[42] Lake D, Corrêa SAL, Müller J (2016). Negative feedback regulation of the ERK1/2 MAPK pathway. Cell Mol Life Sci.

[43] Caunt SM, CJ. K.  (2012). Dual-specificity MAP kinase phosphatases (MKPs): shaping the outcome of MAP kinase signalling. FEBS J.

[44] Gröschl B, Bettstetter M, Giedl C, Woenckhaus M, Edmonston T, Hofstädter F (2013). Expression of the MAP kinase phosphatase DUSP4 is associated with microsatellite instability in colorectal cancer (CRC) and causes increased cell proliferation. Int J cancer.

[45] Emma MR, Iovanna JL, Bachvarov D, Puleio R, Loria GR, Augello G, et al. NUPR1, a new target in liver cancer: Implication in controlling cell growth, migration, invasion and sorafenib resistance. Cell Death Dis. 2016;7. 10.1038/cddis.2016.175 PMC5143401 27336713

[46] Wang C, Che L, Hu J, Zhang S, Jjang L, Latte G (2016). Activated mutant forms of PIK3CA cooperate with RasV12 or c-Met to induce liver tumour formation in mice via AKT2/mTORC1 cascade. Liver Int.

[47] Lu X, Peng B, Chen G, Pes MG, Ribback S, Ament C (2021). YAP Accelerates Notch-Driven Cholangiocarcinogenesis via mTORC1 in Mice. Am J Pathol.

[48] Fan B, Malato Y, Calvisi DF, Naqvi S, Razumilava N, Ribback S (2012). Cholangiocarcinomas can originate from hepatocytes in mice. J Clin Invest.

[49] Smith GS, Walford RL, Mickey MR (1973). Lifespan and incidence of cancer and other diseases in selected long-lived inbred mice and their F 1 hybrids. J Natl Cancer Inst.

[50] Calvisi DF, Ladu S, Conner EA, Seo D, Hsieh J-T, Factor VM (2011). Inactivation of Ras GTPase-activating proteins promotes unrestrained activity of wild-type Ras in human liver cancer. J Hepatol.

[51] Brown WS, McDonald PC, Nemirovsky O, Awrey S, Chafe SC, Schaeffer DF (2020). Overcoming Adaptive Resistance to KRAS and MEK Inhibitors by Co-targeting mTORC1/2 Complexes in Pancreatic Cancer. Cell reports Med.

[52] Seternes Andrew M.; Keyse, Stephen M. OMK. Dual-specificity MAP kinase phosphatases in health and disease. Biochim Biophys acta Mol cell Res. 2018;1866:124–43. 10.1016/j.bbamcr.2018.09.002 PMC6227380 30401534

[53] Gutierrez-Prat N, Zuberer HL, Mangano L, Karimaddini Z, Wolf L, Tyanova S, et al. DUSP4 protects BRAF- and NRAS-mutant melanoma from oncogene overdose through modulation of MITF. Life Sci alliance. 2022;5. 10.26508/lsa.202101235 PMC9113946 35580987

[54] Hijiya N, Tsukamoto Y, Nakada C, Tung Nguyen L, Kai T, Matsuura K (2016). Genomic Loss of DUSP4 Contributes to the Progression of Intraepithelial Neoplasm of Pancreas to Invasive Carcinoma. Cancer Res.

[55] Ichimanda M, Hijiya N, Tsukamoto Y, Uchida T, Nakada C, Akagi T (2018). Downregulation of dual-specificity phosphatase 4 enhances cell proliferation and invasiveness in colorectal carcinomas. Cancer Sci.

