# Supplementary material for: Identification of DUSP4/6 overexpression as a potential rheostat to NRAS-induced hepatocarcinogenesis
Source: BMC Cancer. 2023 Nov 9;23:1086. doi: 10.1186/s12885-023-11577-9 (PMC10636894; doi:10.1186/s12885-023-11577-9)

Figure S1

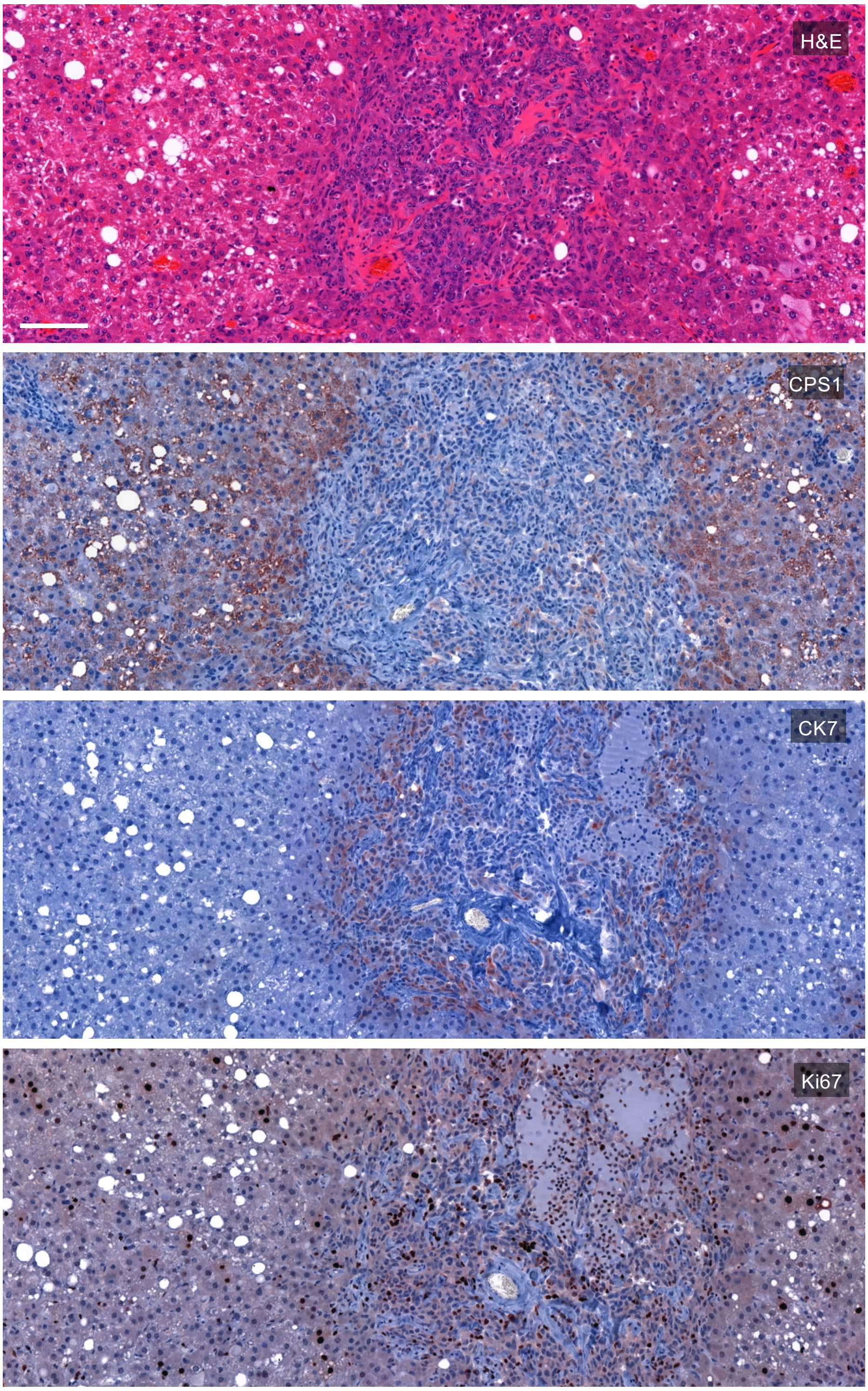

Figure S1. Focal cholangiocellular differentiation within NRAS induced HCC

Tumour with overall HCC morphology and central focus of cholangiocellular differentiation as indicated by positivity for CK7 and reduction of hepatocellular marker CPS1. The high proliferation index within this region confirms its neoplastic nature. Representative image of an occasional observation. *Scale bar:* 100 µm.

Figure S2

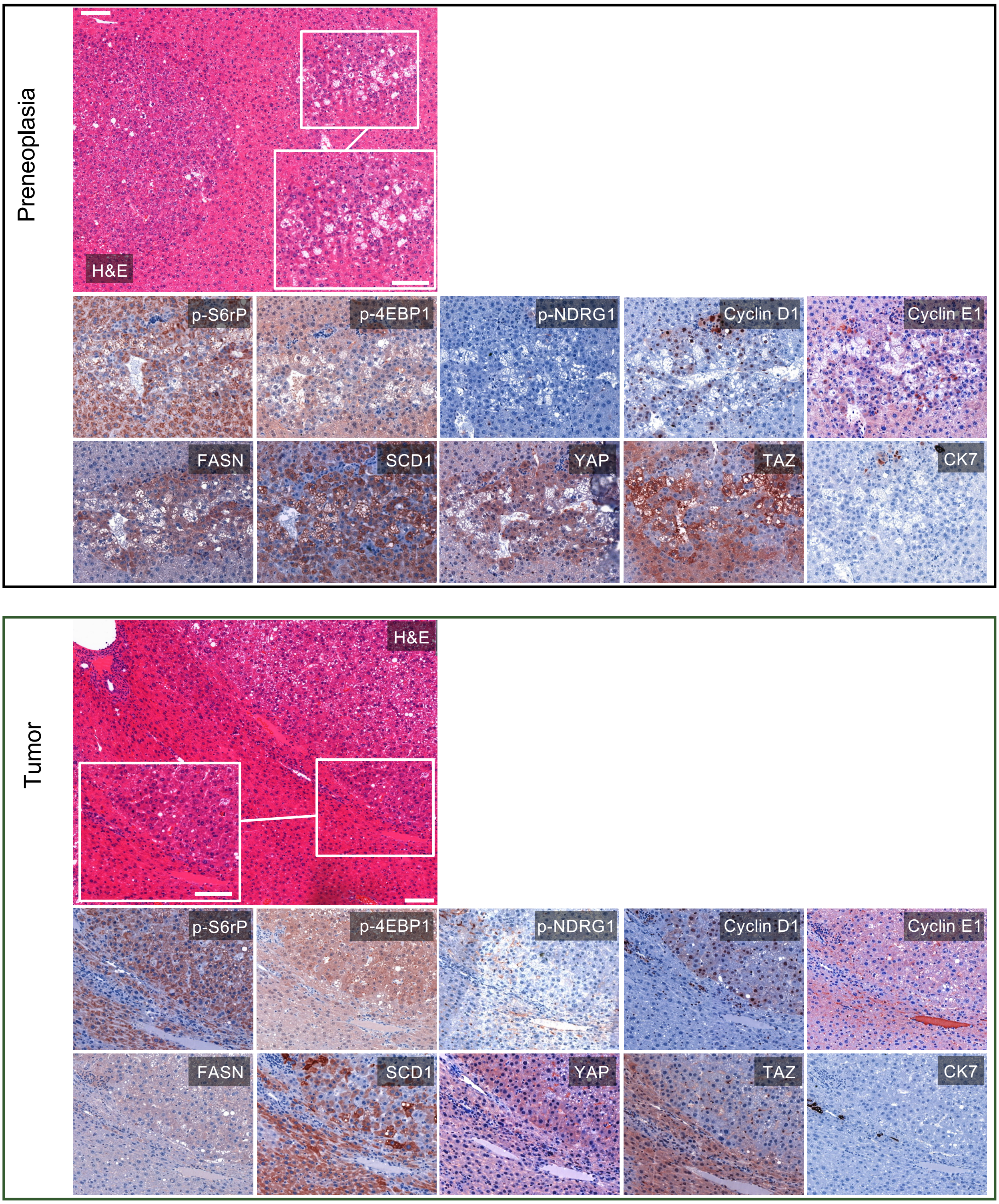

Figure S2. Extended immunohistochemical analysis of *NRAS*^{G12V} induced neoplastic lesions

Preneoplastic lesions (upper panel) and a tumour (lower panel) analysed with the indicated antibodies. *Scale bar:* 100 µm / inset 50 µm.

Figure S3

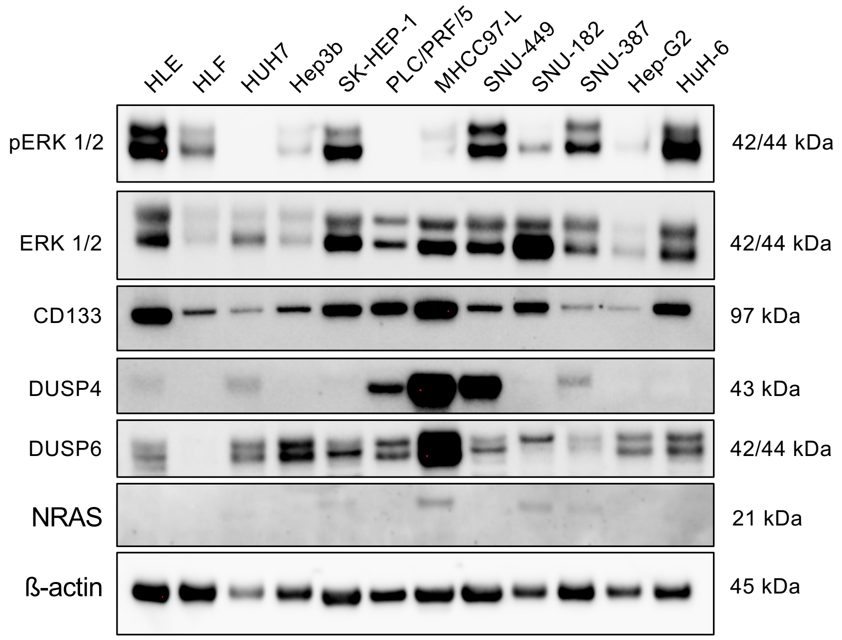

Figure S3. Western blots of selected target proteins in human HCC cell lines. Western blot analysis of indicated HCC cell lines. Molecular weights of observed bands are marked on the right. Note that an *NRAS* Q61K mutation is present in SNU-387 and Hep-G2 carries an *NRAS* Q61L mutation, while the other cell lines are *NRAS* wildtype according to the Cancer Cell Line Encyclopedia (Broad, 2019). Cropped images of Western Blots are shown. The full images of the hybridised cut membranes are included in Figure S7.

Figure S4

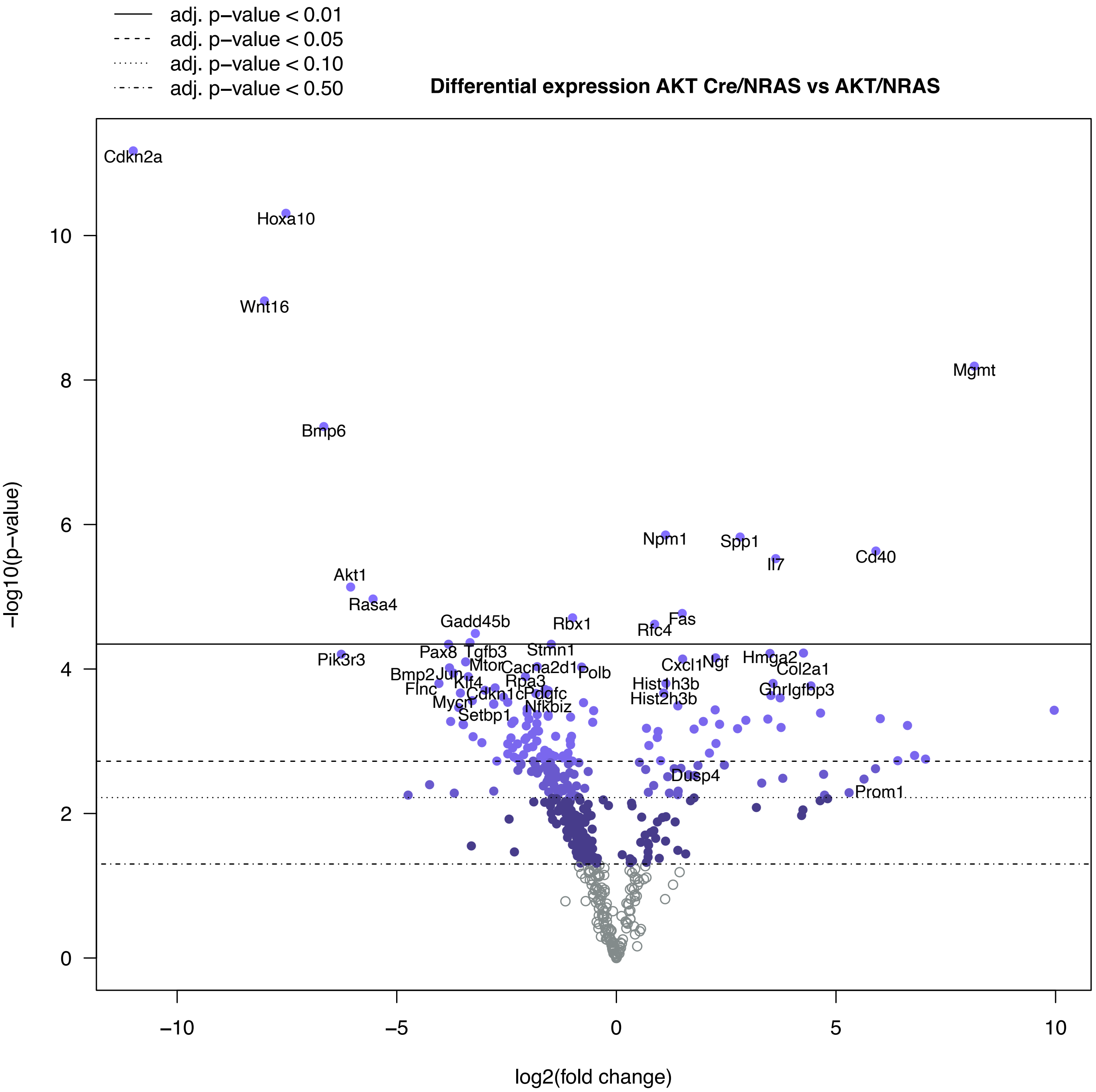

Figure S4. NanoString® gene expression analysis of AKT Cre/NRAS versus AKT/NRAS mouse liver cancer cell lines

Volcano plot of differentially expressed genes obtained from NanoString® mRNA measurement using the mouse Pan Cancer pathway panel®.

Figure S5

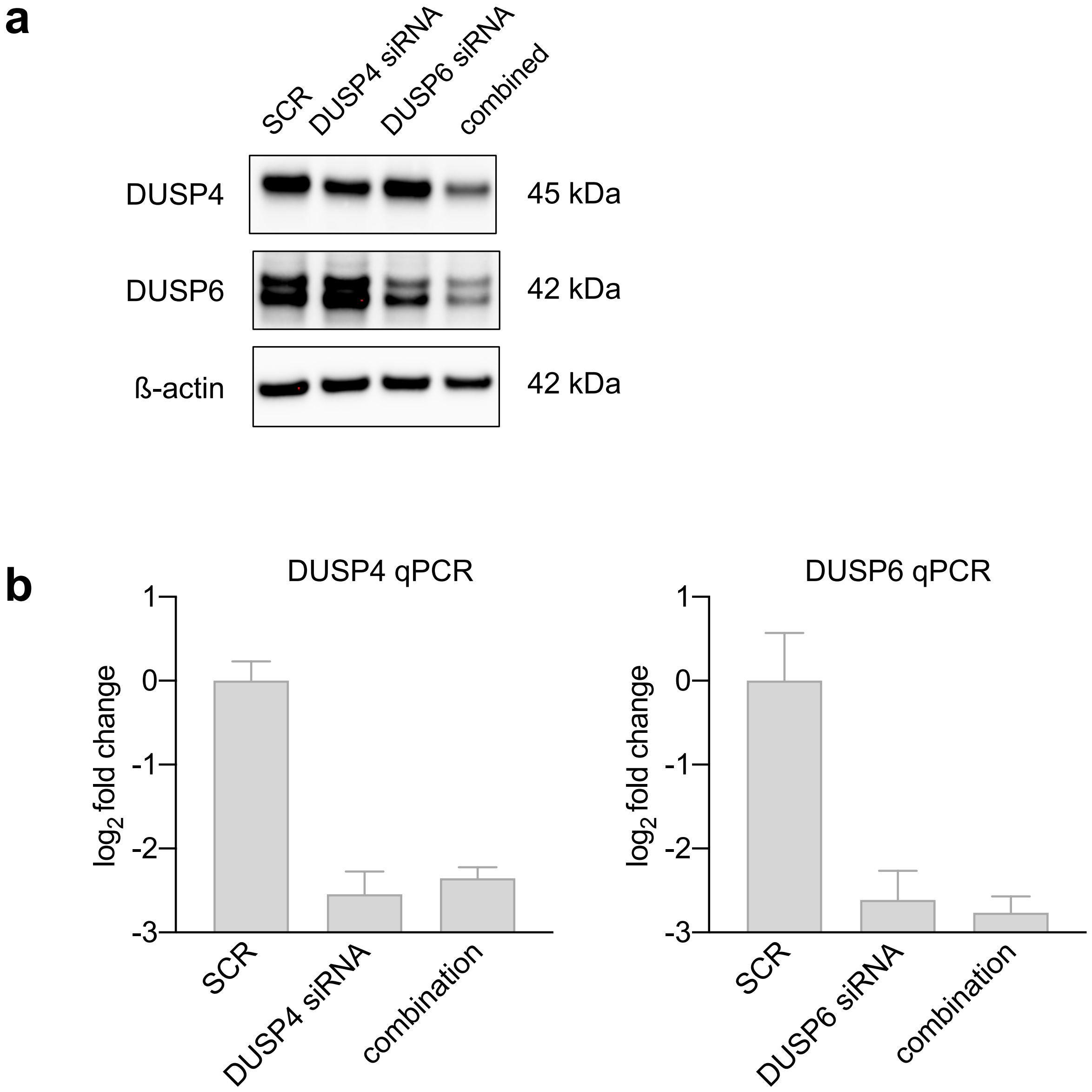

Figure S5. Confirmation of silencing effectiveness for xCELLigence® proliferation experiments

(**a**) Western blot analysis of AKT Cre/ NRAS cell line treated with indicated siRNA or SCR. Molecular weights of observed bands are marked on the right. Cropped images of Western Blots are shown. The full images of the hybridised cut membranes are included in Figure S7.

(**b**) Quantitative real-time PCR analyses of PLC treated with indicated siRNA or SCR. Taqman® probes for DUSP4 on left and DUSP6 on right. Error bars indicate 95% confidence interval.

Figure S6

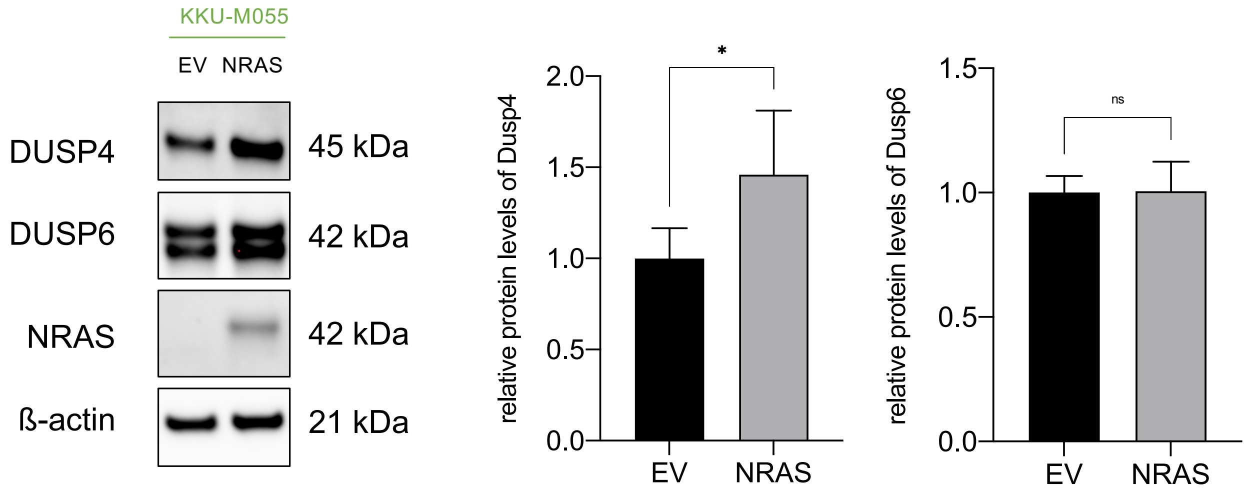

Figure S6. NRAS transfection to CCA cell line

Representative Western blot analysis of $NRAS^{G12V}$ transfection to RAS wild type CCA cell line KKU-M055. Molecular weights of observed bands are marked on the right. Quantification of protein levels of DUSP4 and DUSP6 from 2 repeats with 3 replicates each on the right. Mean with standard deviation. Asterisk indicates $p < 0.05$ (Mann-Whitney-U Test). Cropped images of Western Blots are shown. The full images of the hybridised cut membranes are included in Figure S7.

Figure S7_1

Corresponds to Figure 2*b*

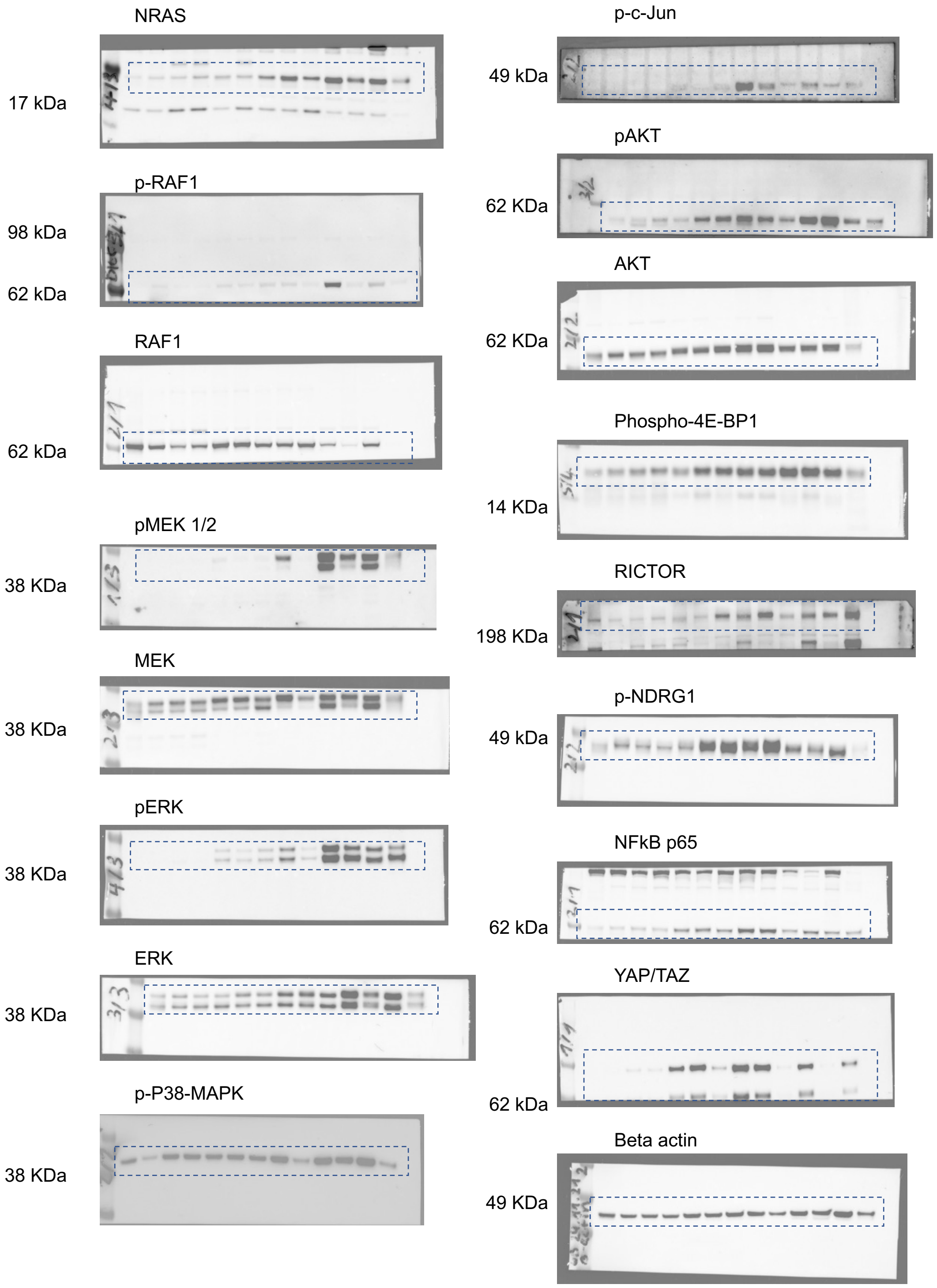

Figure S7. Uncropped Western blots

Uncropped pictures of all Western Blots as shown in the article's figures, including all data from repeats and replicates. Antibody hybridisation was performed on already cut membranes to economise on resources. Occasional lateral cropping means that other samples have been run on the same blot, which are irrelevant to this paper and its conclusions.

Figure S7_2

Corresponds to Figure 4*c*

DUSP4

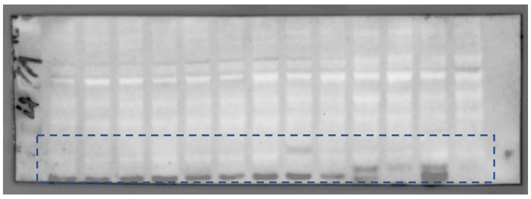

38 kDa

DUSP6

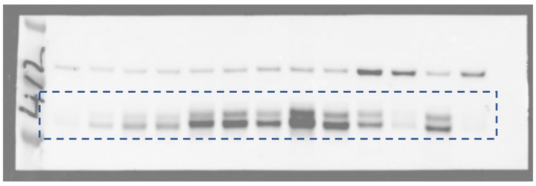

38 kDa

CD133

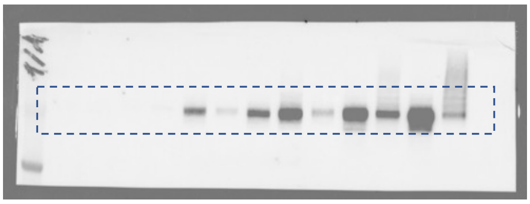

98 kDa

Beta actin

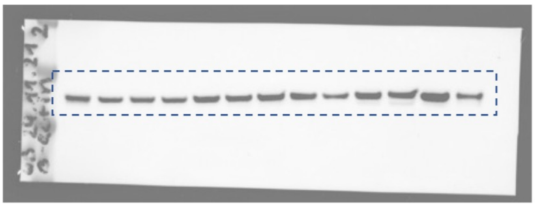

49 KDa

Figure S7_3

Corresponds to Figure 5*a*

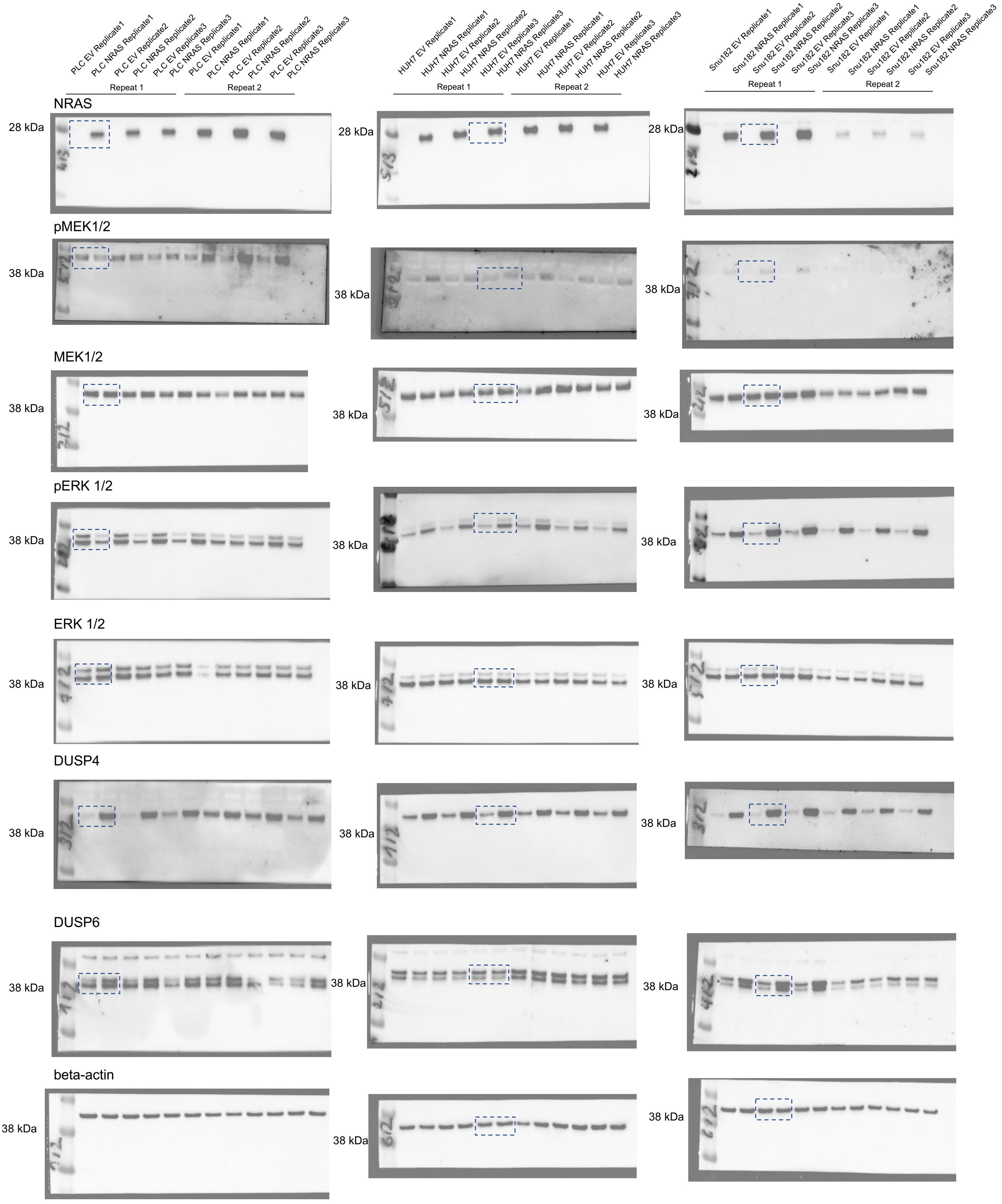

Figure S7_4

Corresponds to Figure 5*b*

1 DMSO 2 µM 30 min
2 TPA 0.5 µM 30 min
3 TPA 2 µM 30 min
4 DMSO 2 µM 60 min
5 TPA 0.5 µM 60 min
6 TPA 2 µM 60 min

PLC **HUH-7** **SNU-182**

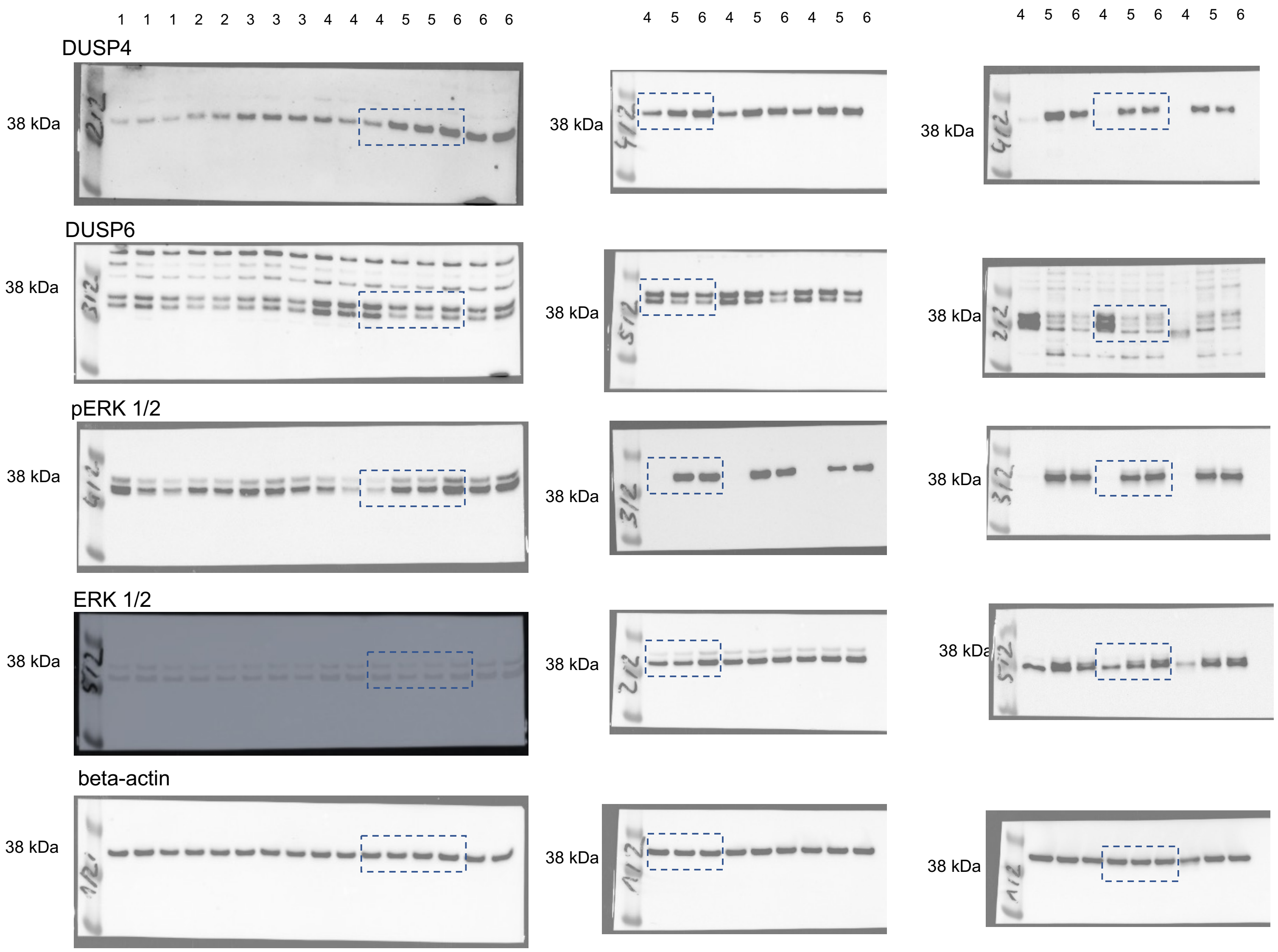

Figure S7_5

Corresponds to Figure 5*c*

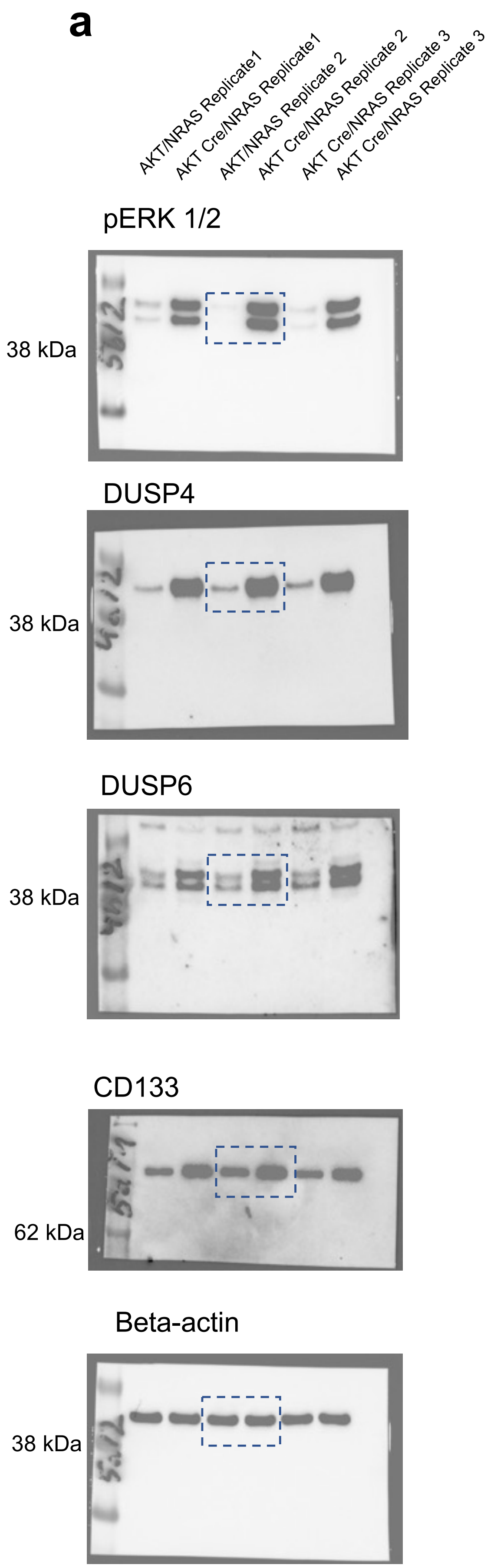

Corresponds to Figure 5*d*

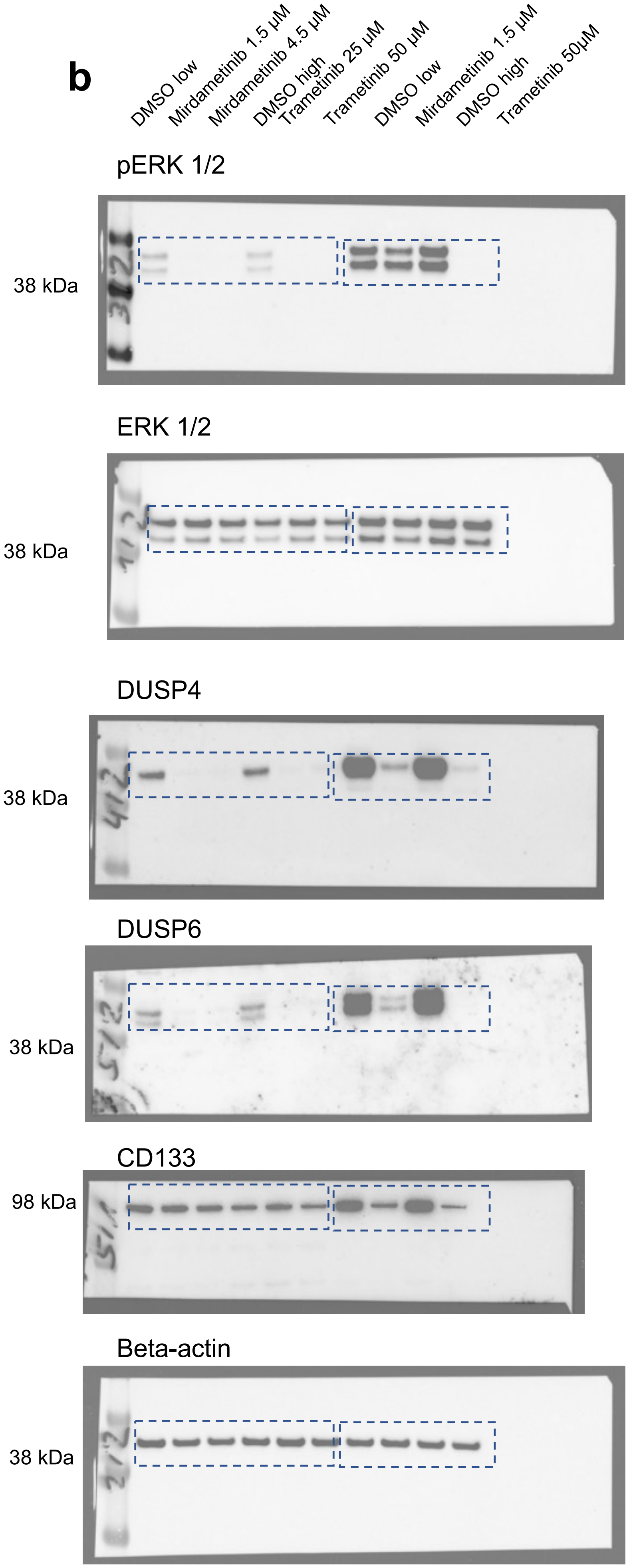

Figure S7_6

Corresponds to Figure 6*a*

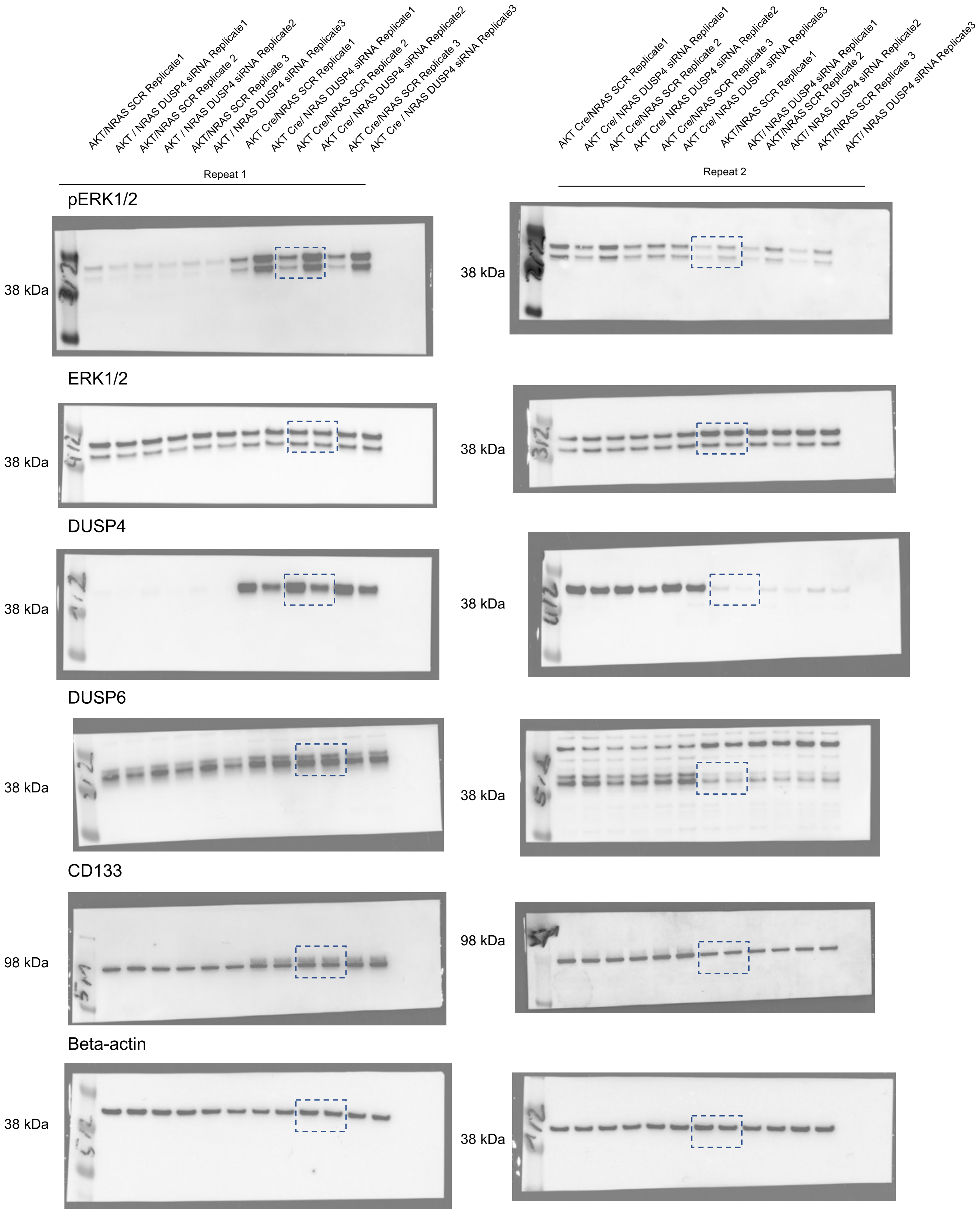

Figure S7_7 Corresponds to Figure 7*a*

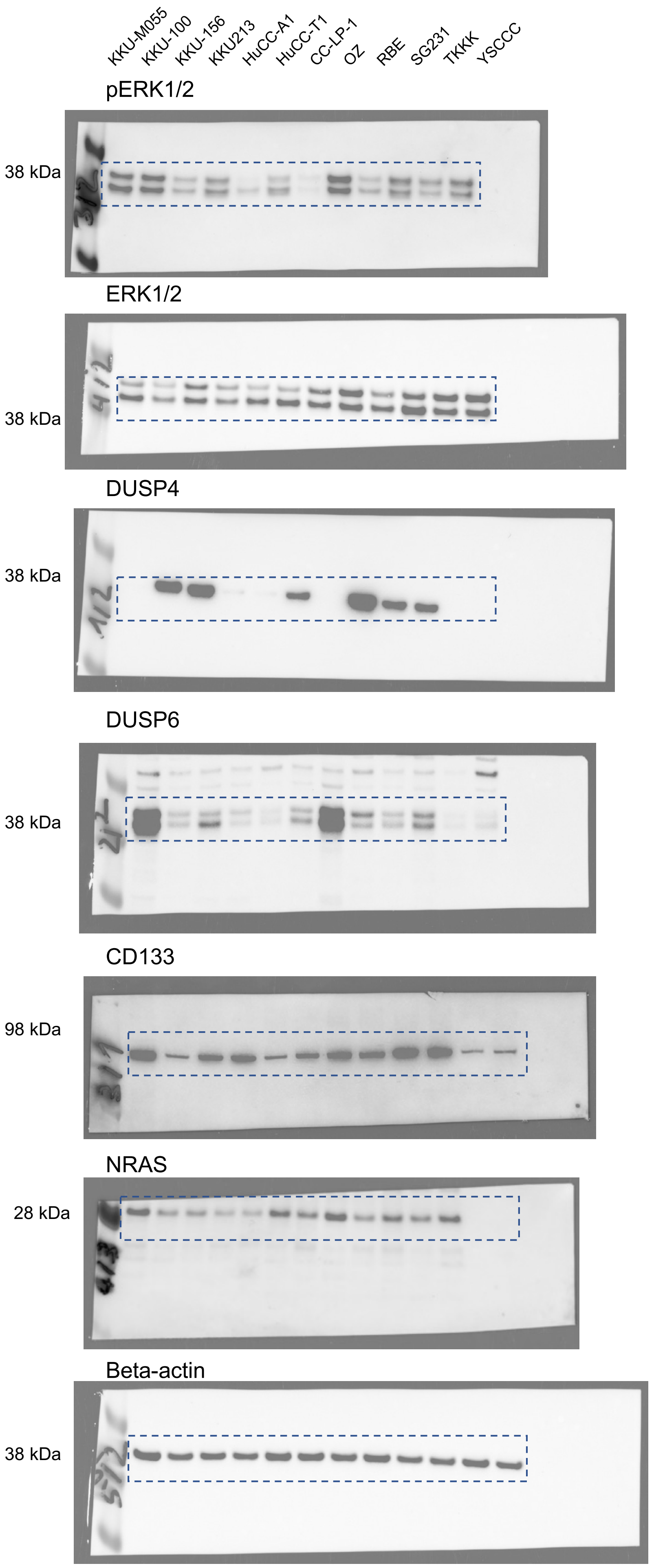

Figure S7_8

Corresponds to Figure S3

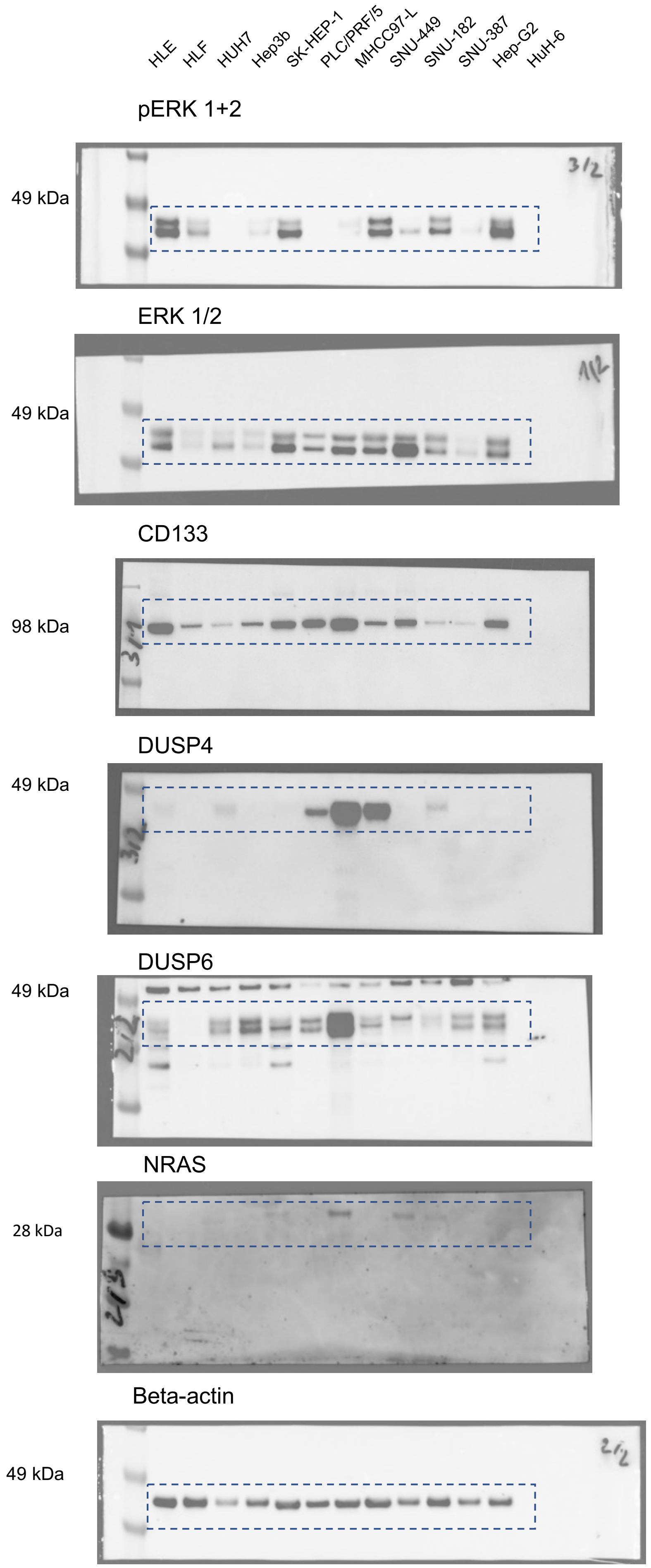

Figure S7_9

Corresponds to Figure S5*a*

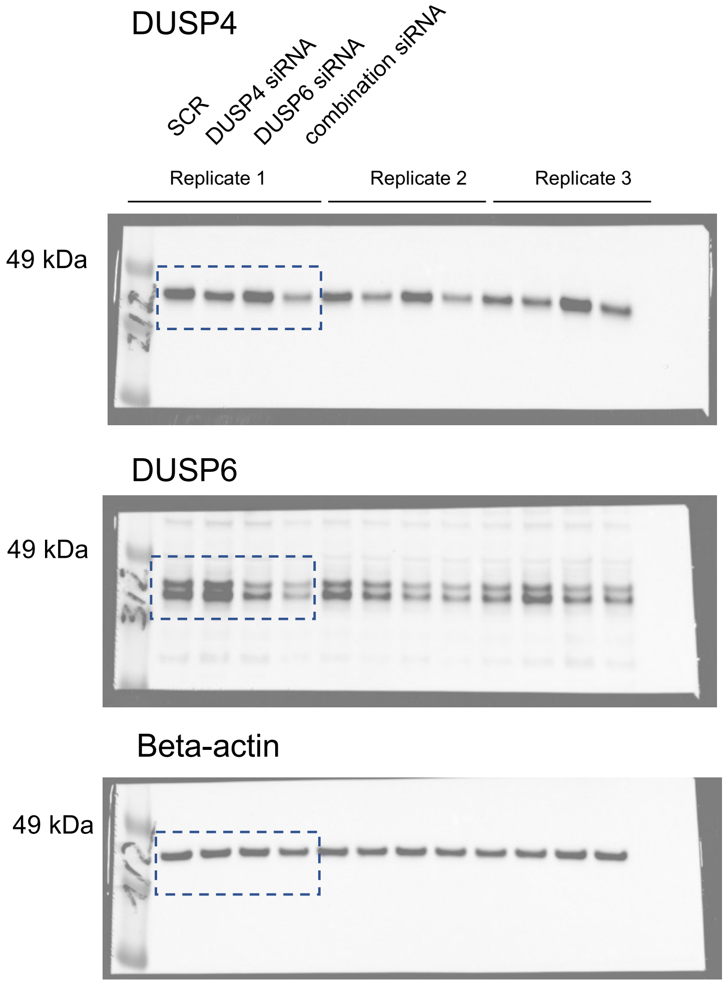

Figure S7_10 Corresponds to Figure S6

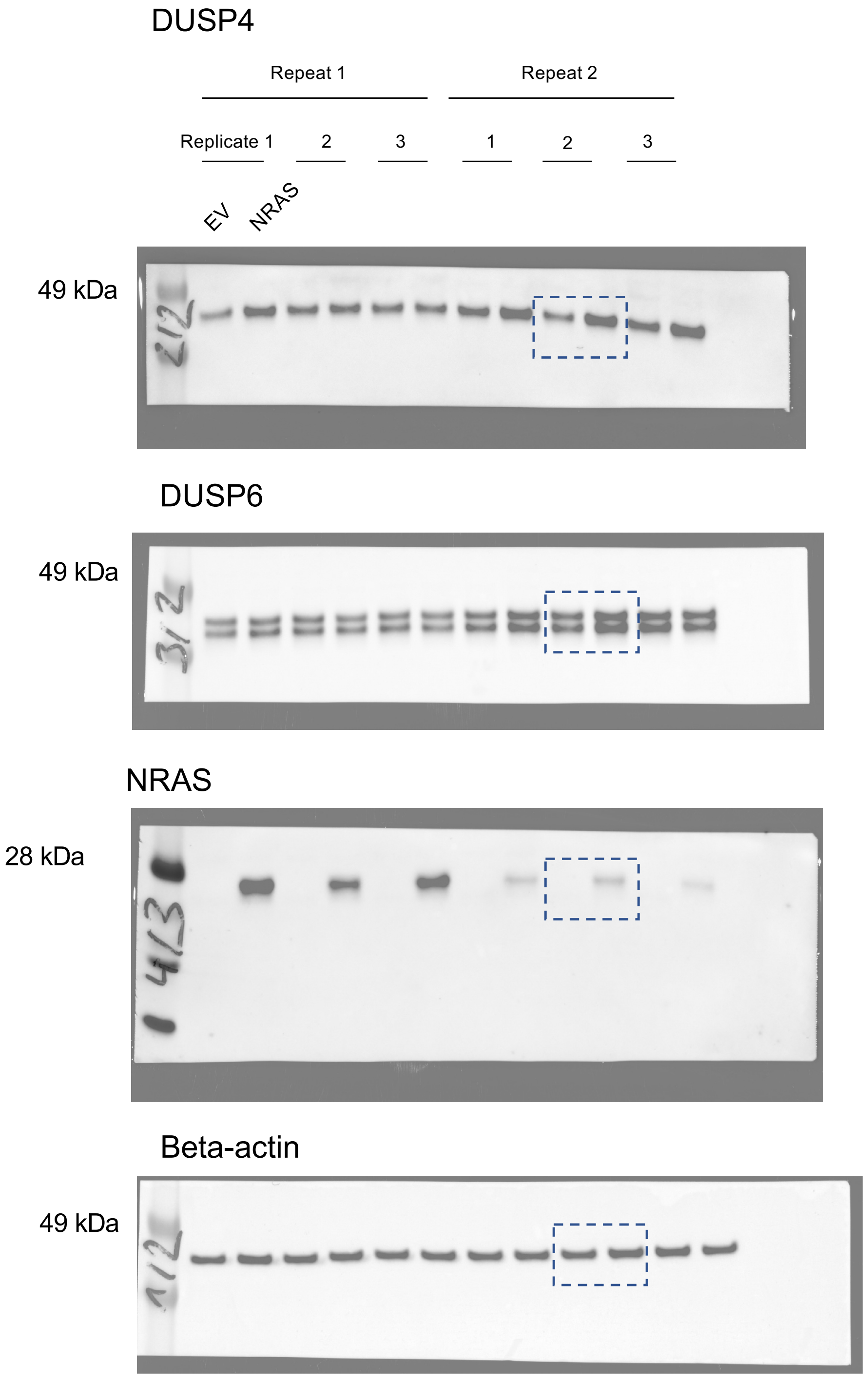

Supplement: Supplementary file 2 — Additional file 2: Supplementary Figures. [file 12885_2023_11577_MOESM2_ESM.pdf]
