# Supplementary material for: Identification of DUSP4/6 overexpression as a potential rheostat to NRAS-induced hepatocarcinogenesis
Source: BMC Cancer. 2023 Nov 9;23:1086. doi: 10.1186/s12885-023-11577-9 (PMC10636894; doi:10.1186/s12885-023-11577-9)
Supplement: Supplementary file 3 — Additional file 3: Supplementary Tables. [file 12885_2023_11577_MOESM3_ESM.pdf]

Supplementary Tables

Supplementary Table 1: Overview of experimental groups and timepoints

	1	2	3	4	5	6	7	8
	Experimental groups		Control groups (previously reported in [18])					
Strain (*RASSF1A*-KO or WT)	KO	WT	KO	WT	KO	WT	KO	WT
Injected construct	$NRAS^{G12V}$	$NRAS^{G12V}$						
1x PBS injection			X	X				
EV					X	X		
untreated							X	X
Animals per timepoint (*N*)								
1 week	10	10	5	5	5	5	5	5
1 month	10	10	5	5	5	5	5	5
3 months	10	10	5	5	5	5	5	5
6 months	10	10	5	5	5	5	5	5
9 months	10	10	5	5	5	5	5	5
12 months	10	10	5	5	5	5	5	5
Sum of animals per experimental group	60	60	30	30	30	30	30	30
Sum of all animals	300							

Supplementary Table 2: Antibodies used for immunohistochemistry and Western blots

Name	Dilution	Company	Catalogue number
**Primary antibodies**			
AKT (pan) (C67E7) Rabbit mAb	WB: 1:1000	Cell Signaling Technology, Inc., Cambridge, UK	4691S
Anti-beta Actin antibody [mAbcam 8226] - Loading Control (HRP)	WB: 1:5000	Abcam plc., Cambridge, UK	ab8226
Anti-CD133 antibody – Stem Cell Marker	WB: 1:1000<br>IHC 1:100	Abcam plc., Cambridge, UK	ab16518
Anti-NRAS antibody	WB: 1:500	Abcam plc., Cambridge, UK	ab77392
Anti-phospho-mTOR (Ser2448) Antibody	WB: 1:333	Merck KGaA, Darmstadt, Germany	09-213
C/EBPα (D56F10) XP® Rabbit mAb	WB: 1:1000<br>IHC: 1:100	Cell Signaling Technology, Inc., Cambridge, UK	8178S
Cyclin D1 (E3P5S) XP® Rabbit mAb	IHC: 1:100	Cell Signaling Technology, Inc., Cambridge, UK	55506S
Cyclin E1 Polyclonal Antibody	IHC: 1:100	Proteinech Group, Inc., Rosemont, USA	11554-1-AP
mTOR Antibody	WB: 1:333	Merck KGaA, Darmstadt, Germany	04-385
N-Ras Antikörper (C-20)	WB: 1:50<br>IHC: 1:100	Santa Cruz Biotechnology, Inc., Dallas, USA	sc-519
NF-κB p65 (D14E12) XP® Rabbit mAb	WB: 1:500	Cell Signaling Technology, Inc., Cambridge, UK	8242S
Osteopontin polyclonal Antibody	WB: 1:500	Proteinech Group, Inc., Rosemont, USA	22952-1-AP
p-Raf-1 (Ser259)-R	WB: 1:200	Santa Cruz Biotechnology, Inc., Dallas, USA	sc-21833
p44/42 MAPK (ERK 1/2) (137F5) Rabbit mAb	WB: 1:1000	Cell Signaling Technology, Inc., Cambridge, UK	4695S
Phospho-4E-BP1 (Thr37/46) (236B4) Rabbit mAb	WB: 1:1000<br>IHC: 1:300	Cell Signaling Technology, Inc., Cambridge, UK	2855
Phospho-AKT (Ser473) (D9E) XP® Rabbit mAb	WB: 1:1000<br>IHC: 1:50	Cell Signaling Technology, Inc., Cambridge, UK	4060
Phospho-c-Jun (Ser63) II Antibody	WB: 1:1000	Cell Signaling Technology, Inc., Cambridge, UK	9261S
Phospho-c-Jun (Ser73) (D47G9) XP® Rabbit mAb	WB: 1:1000	Cell Signaling Technology, Inc., Cambridge, UK	3270S
Phospho-MEK1/2 (Ser217/221) (E4M5C) Rabbit mAb	WB: 1:1000<br>WB: 1:500<br>IHC: 1:100	Cell Signaling Technology, Inc., Cambridge, UK	86128S
Phospho-NDRG1 (Thr346) (D98G11) XP® Rabbit mAb	WB: 1:500<br>IHC: 1:500	Cell Signaling Technology, Inc., Cambridge, UK	5482S
Phospho-NF-κB p65 (Ser276) Antibody	WB: 1:500	Cell Signaling Technology, Inc., Cambridge, UK	3037S
Phospho-p44/42 MAPK (ERK1/2) (Thr202/Tyr204) (D13.14.4E) XP® Rabbit mAb	WB: 1:1000<br>IHC: 1:100	Cell Signaling Technology, Inc., Cambridge, UK	4370S
Phospho-S6 Ribosomal Protein (Ser235/236) (2F9) Rabbit mAb	WB: 1:1000<br>IHC: 1:100	Cell Signaling Technology, Inc., Cambridge, UK	4856S
Rabbit anti-FASN Antibody	WB: 1:100<br>IHC: 1:100	Bethyl Laboratories, Inc., Montgomery, USA	A301-323A
Rabbit anti-Ki-67 IHC Antibody	IHC: 1:100	Bethyl Laboratories, Inc., Montgomery, USA	ICH-00375
RAF1 Antibody	WB: 1:500	LifeSpan BioSciences, Inc, Seattle; USA	LS-C49537
Ral B Antibody (R-19)	WB: 1:200	Santa Cruz Biotechnology, Inc., Dallas, USA	sc-1531
RALA Polyclonal Antibody	WB: 1:333	Thermo Fisher Inc. Waltham, USA	OSR00268W
RAS (E8N8L) XP® Rabbit mAb	IHC: 1:100	Cell Signaling Technology, Inc., Cambridge, UK	67648S

Recombinant Ant-CPS1 antibody [EPR7493-3]	IHC: 1:100	Abcam plc., Cambridge, UK	ab129076
Recombinant Anti-c-Myc antibody [Y69]	WB: 1:1000	Abcam plc., Cambridge, UK	ab32072
Recombinant Anti-CD133 antibody [EPR24348-38]	WB: 1:500<br>IHC: 1:200	Abcam plc., Cambridge, UK	ab271092
Recombinant Anti-Cytokeratin 7 antibody [EPR17078] – Cytoskeleton Marker	IHC: 1:400	Abcam plc., Cambridge, UK	ab181598
Recombinant Anti-DUSP4 antibody [EPR19881]	WB: 1:500<br>WB: 1:200	Abcam plc., Cambridge, UK	ab216576
Polyclonal Rabbit anti-Human DUSP4 / MKP2 Antibody	IHC: 1:100	Lifespan Bioscience, Seattle, USA	LS-B14575
Recombinant Anti-DUSP6 antibody [EPR129Y]	WB: 1:500<br>IHC: 1:100	Abcam plc., Cambridge, UK	ab76310
Recombinant Anti-EpCAM antibody [EPR20533-63]	IHC: 1:100	Abcam plc., Cambridge, UK	ab221552
Recombinant Anti-MEK1 + MEK2 antibody [EPR16667]	WB: 1:1000	Abcam plc., Cambridge, UK	ab178876
Recombinant Anti-Osteopontin antibody [RM1018]	WB: 1:1000<br>IHC: 1:200	Abcam plc., Cambridge, UK	ab283656
RICTOR Antibody	WB: 1:1000	Bethyl Laboratories, Inc., Montgomery, USA	A300-459A
SCD1 (C12H5) Rabbit mAb	WB: 1:1000<br>IHC: 1:100	Cell Signaling Technology, Inc., Cambridge, UK	2794S
YAP/TAZ (D24E4) Rabbit mAb	WB: 1:1000	Cell Signaling Technology, Inc., Cambridge, UK	8418
**Secondary antibodies**			
Anti-Goat IgG (whole molecule) – Peroxidase antibody produced in rabbit	1:20000	Sigma-Aldrich, Inc., St. Louis, USA	A4174
HRP, Goat Anti-Mouse IgG	1:20000	Abbkine Scientific Co., Ltd., Wuhan, CN	A21010
HRP, Goat Anti-Rabbit IgG	1:20000	Abbkine Scientific Co., Ltd., Wuhan, CN	A21020
N-Histofine® Simple Stain Mouse MAX PO (Rat) Anti-Rat	1:20000	Nichirei Biosciences Inc., Tokyo, Japan	414311F
ZytoChem Plus HRP-Polymer anti-Rabbit	1:20000	Zytomed Systems GmbH, Berlin, Germany	ZUC032-100

Supplementary Table 3: Taqman® probes

Name	Company	Catalogue number
TaqMan™ Actb mouse VIC 20x Mix	Thermo Fisher Inc. Waltham, USA	Mm00607939_s1
TaqMan™ DUSP4 human 20x Mix	Thermo Fisher Inc. Waltham, USA	Hs01027785_m1
TaqMan™ DUSP4 mouse FAM 20x Mix	Thermo Fisher Inc. Waltham, USA	Mm00723761_m1
TaqMan™ DUSP6 human 20x Mix	Thermo Fisher Inc. Waltham, USA	Hs04329643_s1
TaqMan™ DUSP6 mouse FAM 20x Mix	Thermo Fisher Inc. Waltham, USA	Mm00518185_m1
TaqMan™ GAPDH human VIC 60x Mix	Thermo Fisher Inc. Waltham, USA	Hs02786624_g1
TaqMan™ NUPR1 mouse FAM 20x Mix	Thermo Fisher Inc. Waltham, USA	Mm00498104_m1
