# Supplementary material for: Identification of DUSP4/6 overexpression as a potential rheostat to NRAS-induced hepatocarcinogenesis
Source: BMC Cancer. 2023 Nov 9;23:1086. doi: 10.1186/s12885-023-11577-9 (PMC10636894; doi:10.1186/s12885-023-11577-9)
Supplement: Supplementary file 4 — Additional file 4: Supplementary Methods. [file 12885_2023_11577_MOESM4_ESM.pdf]

Supplementary methods

Histology and immunohistochemistry

Two board-certified pathologists (equivalent to the status “Facharzt” in the German medical system) and liver experts were responsible for the histological assessment of the specimens (K.U., K.E.). For tissue processing, we followed our standard institutional guidelines, which have been reported before [1]. For immunohistochemistry, 5 µm sections were cut on a Scientific Rotary Microtome Microtom HM 340 (Thermo Fisher Inc. Waltham, USA). Deparaffinization was achieved with a series of xylene and gradient alcohols to water. Slides were cooked in a Decloaking ChamberTM NxGen (Biocare Medical, LLC., Pacheco; US) at 110°C for 8 min within either 10 mM sodium citrate (pH 6.0) or Tris EDTA (pH 9.0). Next, the slides were treated with 1× Dako Peroxidase-Blocking Solution® (cat S2023; Agilent Technologies Inc., Santa Clara, CA) for 15 min. Subsequently, the primary antibody (Supplementary Table 2) was applied in Dako Antibody Diluent® (cat. S2022, Agilent Technologies Inc.). An overnight incubation in a humidity chamber at room temperature followed. After applying Dako washing solution® (cat. S3006, Agilent Technologies Inc.), the secondary antibodies (Supplementary Table 2) were administered for 60 min at room temperature. Two washes in Dako washing solution® followed. For chromogenic reactions, ImmPACT® NovaRED® Substrate Kit ® (Agilent Technologies Inc.) was applied for 10-15 minutes at room temperature. Next, slides were submerged in Mayer’s hemalum 8 times for 10 s and kept under running tap water for 5-10 minutes. Coverslips were added after final dehydration in isopropanol and xylene.

The slide scanner Pannoramic 1000 Flash RX® (Sysmex Europe SE, Norderstedt, Germany) was used for image acquisition choosing the 20x objective. Virtual images

from this stitched representation were collected for demonstration purposes using the software CaseViewer version 2.4 (Sysmex Europe SE).

Protein isolation and Western blotting

Protein extraction from mouse liver samples was achieved with T-Per lysis buffer (Thermo Fisher Scientific) in Navy Eppendorf® Lysis Cups (Eppendorf, Hamburg, Germany) on ice. Bullet Blender® Storm (Next Advance, Inc., Troy, USA) was used for effective homogenization. After shaking for 60 minutes at 1000 rpm and 4°C, centrifugation was performed for 5 minutes (>12.000 g, 4°C). The supernatant was transferred and centrifuged for 30 minutes (>20,000 g, 4°C). This supernatant was used for the subsequent analyses. For protein isolation from cell pellets, the T-Per lysis buffer with HALT protease and phosphatase inhibitor (Thermo Fisher Scientific) was incubated for 30 minutes on ice, and samples were regularly vortexed. Centrifugation for 30 minutes (>20,000 g, 4°C) followed, and the supernatant was collected. We assessed the concentrations of total proteins with the Bradford Protein Assay [2]. Serial dilutions of BSA between 500 and 31.25 µg/µl were employed to yield a standard curve for linear regression. Quantification was performed with the FLUOstar® Omega microplate reader (BMG LABTECH GmbH, Ortenberg, Germany).

Sodium dodecyl sulfate-polyacrylamide gel electrophoresis was used for Western blotting. 2.5 µg of total proteins were loaded onto BoltTM 4-12% Bis-Tris Plus gels (Thermo Fisher Scientific) at 120-150 V for 60-75 min. Gels were incubated in 10% ethanol for dehydration before blotting the proteins to a membrane using the BlotTM 2 Gel Transfer Device (Thermo Fisher Scientific). Membranes were stained in Ponceau S solution and then placed in EveryBlot Blocking Buffer (Bio-Rad Laboratories, Inc.; Hercules, CA) for 15-20 minutes at room temperature. Primary antibodies (Supplementary Table 2) were incubated overnight at 4°C in the blocking buffer. The

next day, membranes were washed in Tris Buffered Saline with Tween® (Cell Signaling Technology, Inc.) for 5 minutes. Afterward, the secondary antibody was applied at room temperature for 1 hour. The membranes were washed three times with Tris Buffered Saline with Tween®. The chemiluminescent signal was visualized by Clarity Max™ Western ECL Substrate (Bio-Rad Laboratories) on a ChemiDoc™ MP Imaging System (Bio-Rad Laboratories). For quantitative analysis of band intensities, version 6.1 of the software ImageLab (Bio-Rad Laboratories) was used. For quantification, purposed values were normalized to the respective β-actin band intensities.

The membrane fragments hybridised with the respective antibodies are included in Figure S7 with the indication of areas cropped in the figures. Due to the cutting before hybridization, full-length blots cannot be shown. One position of the protein standard is indicated there so that all molecular weights can be inferred. NovexTM SeeBlueTM Plus2 Prestained Standard (Thermo Fisher Inc.) was used as standard. Sometimes blots were cut to be able to probe multiple different proteins.

Nucleic acid extraction and quantitative reverse transcription real-time polymerase chain reaction (qRT-PCR)

Total RNA was collected with the AllPrep® DNA/RNA Mini Kit (Qiagen Sciences Inc., Germantown, USA) and RNeasy® Kit NucleoSpin® RNA Plus Kit (Macherey-Nagel GmbH & Co, Düren, Germany) from pelleted cells or tumorous/ non tumorous liver tissue (after homogenization with the respective lysis buffers included in the above-mentioned kits). Reverse transcription was achieved using the High-Capacity cDNA Reverse Transcription Kit with RNase Inhibitor (Thermo Fisher Scientific). The CFX96TM Real-Time System (Bio-Rad Laboratories, Inc.) was employed. The following protocol was followed: 1.0 µl primer mix, 5 µl TaqMan Universal PCR Master Mix (ThermoFisher Scientific), 0.5 µl Taqman target, 0.17 µl GAPDH Taqman, 1 µl

cDNA (100 ng), and 3.33 µl d2H2O. Cycling conditions were: polymerase activation at 95°C for 2 min, 40 cycles: denaturation at 94°C for 30 s, annealing at 55°C for 30 s, and extension at 72°C for 30 s. The Taqman® probes we utilized are summarized in Supplementary Table 3.

Patient samples

A total of 45 archived CCA samples (time range of surgery 1996 – 2018) had previously been analysed for KRAS mutations by high-resolution melting curve analysis by Alexandra Muggli. The use of archived tissue for research purposes was conducted in accordance with the Declaration of Helsinki and the Bayerisches Krankenhausgesetz. From these the corresponding RNA had been extracted as described above.

References

1. Scheiter A, Evert K, Reibenspies L, Cigliano A, Annweiler K, Müller K, et al. RASSF1A independence and early galectin-1 upregulation in PIK3CA-induced hepatocarcinogenesis: new therapeutic venues. Mol Oncol. 2022;16:1091–118.
2. Bradford MM. A rapid and sensitive method for the quantitation of microgram quantities of protein utilizing the principle of protein-dye binding. Anal Biochem. 1976. https://doi.org/10.1016/0003-2697(76)90527-3.
